# Inactivated Vaccine-Induced SARS-CoV-2 Variant-Specific Immunity in Children

**DOI:** 10.1128/mbio.01311-22

**Published:** 2022-11-16

**Authors:** Jorge A. Soto, Felipe Melo-González, Cristián Gutierrez-Vera, Bárbara M. Schultz, Roslye V. Berríos-Rojas, Daniela Rivera-Pérez, Alejandro Piña-Iturbe, Guillermo Hoppe-Elsholz, Luisa F. Duarte, Yaneisi Vázquez, Daniela Moreno-Tapia, Mariana Ríos, Pablo A. Palacios, Richard Garcia-Betancourt, Álvaro Santibañez, Gaspar A. Pacheco, Constanza Mendez, Catalina A. Andrade, Pedro H. Silva, Benjamín Diethelm-Varela, Patricio Astudillo, Mario Calvo, Antonio Cárdenas, Marcela González, Macarena Goldsack, Valentina Gutiérrez, Marcela Potin, Andrea Schilling, Lorena I. Tapia, Loreto Twele, Rodolfo Villena, Alba Grifoni, Alessandro Sette, Daniela Weiskopf, Rodrigo A. Fasce, Jorge Fernández, Judith Mora, Eugenio Ramírez, Aracelly Gaete-Argel, Mónica L. Acevedo, Fernando Valiente-Echeverría, Ricardo Soto-Rifo, Angello Retamal-Díaz, Nathalia Muñoz-Jofré, PedCoronaVac03CL Study Group,, Xing Meng, Qianqian Xin, Eduardo Alarcón-Bustamante, José V. González-Aramundiz, Nicole Le Corre, María Javiera Álvarez-Figueroa, Pablo A. González, Katia Abarca, Cecilia Perret, Leandro J. Carreño, Susan M. Bueno, Alexis M. Kalergis

**Affiliations:** a Millennium Institute on Immunology and Immunotherapy, Santiago, Chile; b Departamento de Genética Molecular y Microbiología, Facultad de Ciencias Biológicas, Pontificia Universidad Católica de Chile, Santiago, Chile; c Departamento de Ciencias Biológicas, Facultad de Ciencias de la Vida, Universidad Andrés Bello, Santiago, Chile; d Programa de Inmunología, Instituto de Ciencias Biomédicas, Facultad de Medicina, Universidad de Chile, Santiago, Chile; e Departamento de Enfermedades Infecciosas e Inmunología Pediátricas, División de Pediatría, Escuela de Medicina, Pontificia Universidad Católica de Chile, Santiago, Chile; f Instituto de Pediatría, Facultad de Medicina, Universidad Austral de Chile, Valdivia, Chile; g Departamento de Ciencias Médicas, Facultad de Medicina y Odontología, Universidad de Antofagasta, Antofagasta, Chile; h Hospital Dr. Gustavo Fricke, Valparaiso, Chile; i Departamento de Pediatría, Universidad de Valparaíso, Valparaiso, Chile; j Unidad de Infectología Pediátrica, Servicio de Pediatría, Hospital Dr. Sotero del Rio, Santiago, Chile; k Clínica San Carlos de Apoquindo, Red de Salud UC Christus, Santiago, Chile; l Facultad de Medicina, Clínica Alemana, Universidad del Desarrollo, Santiago, Chile; m Departamento de Pediatría y Cirugía Infantil Norte, Hospital Roberto del Río, Santiago, Chile; n Programa de Virología, Instituto de Ciencias Biomédicas, Facultad de Medicina, Universidad de Chile, Santiago, Chile; o Hospital Puerto Montt, Puerto Montt, Chile; p Hospital Exequiel González Cortés, Santiago, Chile; q Facultad de Medicina, Departamento de Pediatría y Cirugía Infantil Campus Sur, Universidad de Chile, Santiago, Chile; r Center for Infectious Disease and Vaccine Research, La Jolla Institute for Immunology (LJI), La Jolla, California, USA; s Department of Medicine, Division of Infectious Diseases and Global Public Health, University of California, San Diego (UCSD), La Jolla, California, USA; t Departamento de Laboratorio Biomédico, Instituto de Salud Pública de Chile, Santiago, Chile; u Laboratorio de Virología Molecular y Celular, Programa de Virología, Instituto de Ciencias Biomédicas, Facultad de Medicina, Universidad de Chile, Santiago, Chile; v Departamento de Biotecnología, Facultad de Ciencias del Mar y de Recursos Biológicos, Universidad de Antofagasta, Antofagasta, Chile; w Departamento de Tecnología Médica, Facultad de Ciencias de la Salud, Universidad de Antofagasta, Antofagasta, Chile; x Sinovac Biotech, Beijing, China; y Faculty of Mathematics, Pontificia Universidad Católica de Chile, Santiago, Chile; z Millennium Nucleus on Intergenerational Mobility: From Modelling to Policy (MOVI), Santiago, Chile; aa Interdisciplinary Laboratory of Social Statistics, Faculty of Mathematics, Pontificia Universidad Católica de Chile, Santiago, Chile; bb Departamento de Farmacia, Facultad de Química y de Farmacia, Pontificia Universidad Católica de Chile, Santiago, Chile; cc Departamento de Endocrinología, Facultad de Medicina, Escuela de Medicina, Pontificia Universidad Católica de Chile, Santiago, Chile; dd Departamento de Pediatría, Clínica Alemana de Santiago, Santiago, Chile; ee Facultad de Medicina y Ciencia, Universidad San Sebastián, Puerto Montt, Chile; NIAID, NIH

**Keywords:** CoronaVac, phase 3 clinical trial, pediatric, SARS-CoV-2, COVID-19, vaccines, variants of concern, immunogenicity, safety

## Abstract

Multiple vaccines against severe acute respiratory syndrome coronavirus 2 (SARS-CoV-2) have been evaluated in clinical trials. However, trials addressing the immune response in the pediatric population are scarce. The inactivated vaccine CoronaVac has been shown to be safe and immunogenic in a phase 1/2 clinical trial in a pediatric cohort in China. Here, we report interim safety and immunogenicity results of a phase 3 clinical trial for CoronaVac in healthy children and adolescents in Chile. Participants 3 to 17 years old received two doses of CoronaVac in a 4-week interval until 31 December 2021. Local and systemic adverse reactions were registered for volunteers who received one or two doses of CoronaVac. Whole-blood samples were collected from a subgroup of 148 participants for humoral and cellular immunity analyses. The main adverse reaction reported after the first and second doses was pain at the injection site. Four weeks after the second dose, an increase in neutralizing antibody titer was observed in subjects relative to their baseline visit. Similar results were found for activation of specific CD4^+^ T cells. Neutralizing antibodies were identified against the Delta and Omicron variants. However, these titers were lower than those for the D614G strain. Importantly, comparable CD4^+^ T cell responses were detected against these variants of concern. Therefore, CoronaVac is safe and immunogenic in subjects 3 to 17 years old, inducing neutralizing antibody secretion and activating CD4^+^ T cells against SARS-CoV-2 and its variants. (This study has been registered at ClinicalTrials.gov under no. NCT04992260.)

## INTRODUCTION

The inactivated severe acute respiratory syndrome coronavirus 2 (SARS-CoV-2) vaccine CoronaVac, developed by Sinovac Life Sciences Co., Ltd. (Beijing, China), has shown favorable safety and immunogenicity results in adults (1–5). In children and adolescents between 3 and 17 years old, this vaccine induces neutralizing antibodies against SARS-CoV-2 after immunization with two doses (6). Similar results have been shown with Pfizer BNT162b2 and Moderna mRNA-1273 in children between 6 and 11 years old and adolescents (7, 8). However, these reports lack a characterization of the cellular immune responses elicited in children and adolescents after immunization and the neutralizing capacity of antibodies against SARS-CoV-2 variants of concern (VOC). Here, we further characterize the immune responses elicited in participants between 3 and 17 years old 4 weeks after the second dose of CoronaVac applied in a 4-week interval (or 0- to 28-day vaccination schedule) in a phase 3 trial in Chile. The present study was generated during an epidemiological period with a high presence of SARS-CoV-2 Delta variant and a low number of cases caused by the Omicron variant. The administration of this vaccine in the participants between 3 and 17 years old is safe and elicits significant levels of both humoral and cellular immunity in adolescents and children against the original strain of SARS-CoV-2 and VOC.

## RESULTS

### Population included in the study.

**FIG 1:**
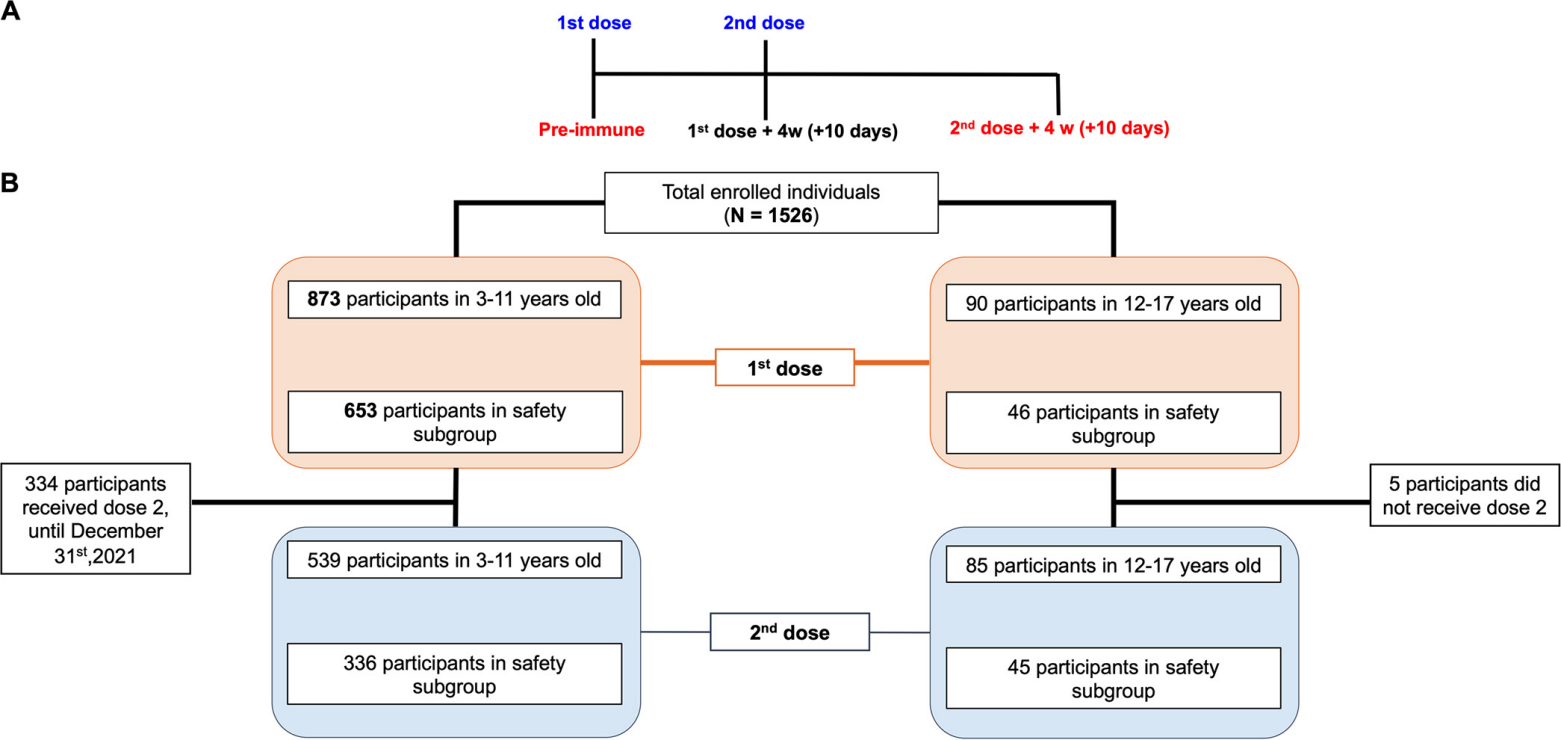
Study profile, enrolled participants, and cohort included in this study from September 10th to December 31st 2021. (A) Timeline of the vaccination schedule and sample collection. Text in red denotes time points at which blood draws occurred. (B) Study profile of subjects that received 1 dose (orange boxes) and 2 doses (light blue boxes) until December 31st 2021 by age and safety group.

Nine hundred sixty-three participants were recruited between September 10th and December 31st; 482 of them were male (51.1%), with an average age of 6.35 years (standard deviation [SD], 3.12 years). The study included children and adolescents 3 to 17 years old who were inoculated with two doses of 3 μg (600 Standard Unit) of CoronaVac in a 4-week interval (0- to 28-day schedule) (Fig. 1A). Figure 1B shows the enrolled population and distribution by age, dose, and safety group.

### Adverse events identified in children and adolescents vaccinated with CoronaVac. (i) Immediate adverse events.

In the 30 min postvaccination, local pain was reported by 3.8% and 1.7% of subjects in the 3- to 11-year age group after the first and second doses, respectively, and 2.2% and 8.2% in the 12- to 17-year age group. Pain adverse event (AE) was statistically significantly higher in adolescents than children after the second dose (*P* = 0.003). The rest of the local AEs were reported in 2% or fewer participants, without age or dose differences, such as pruritus localized in the site of injection of two children of 3 to 11 years old after the first dose (see Table S1 in the supplemental material).

10.1128/mbio.01311-22.7 TABLE S1 Frequency of local and systemic immediate adverse events by dose and age group. Download Table S1, PDF file, 0.03 MB. Copyright © 2022 Soto et al. 2022 Soto et al. https://creativecommons.org/licenses/by/4.0/ This content is distributed under the terms of the Creative Commons Attribution 4.0 International license.

Systemic immediate AEs were reported in less than 1% of the 3- to 11-year age group. Meanwhile, the 12- to 17-year age group reported mainly headache after the first and second doses at 2.2% and 1.2%, respectively. They reported no other systemic AEs after the first dose, and one adolescent reported self-limited pruritus after the second dose (Table S1).

### (ii) Nonimmediate adverse events.

**FIG 2:**
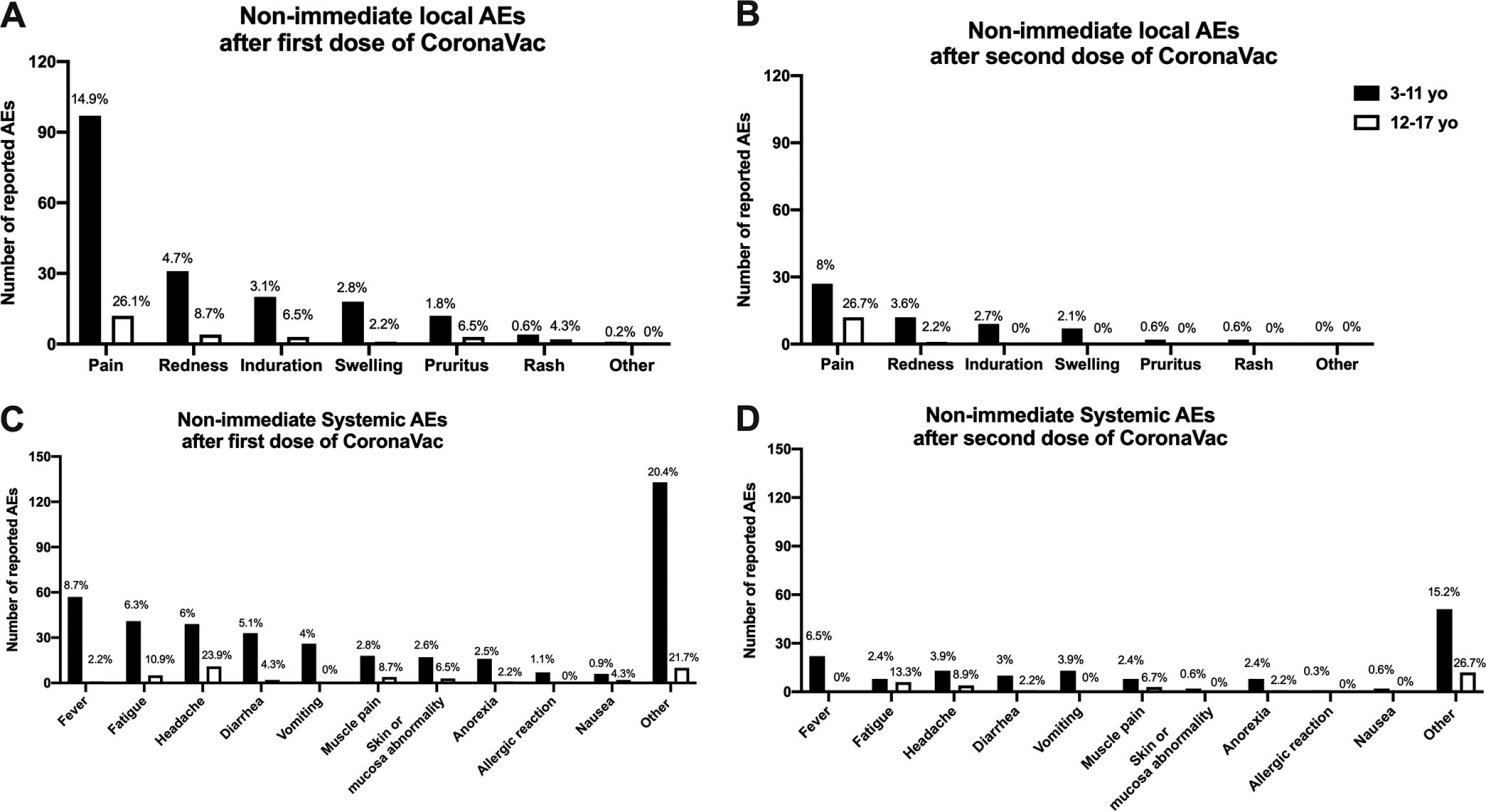
Frequency and duration of nonimmediate local and systemic adverse events by dose and age group. Shown are the total number of nonimmediate local AEs presented by 653 participants in the 3- to 11-year age group and 46 participants in the 12- to 17-year age group after their first dose of CoronaVac (A) or 336 participants in the 3- to 11-year age group and 45 participants in the 12- to 17-year age group after their second dose of CoronaVac (B). (C and D) Total number of systemic AEs presented by 653 participants in the 3- to 11-year age group and 46 participants in the 12- to 17-year age group after the first (C) or the second (D) dose of CoronaVac. Black bars correspond to the 3- to 11-year age group, and white bars correspond to the 12- to 17-year age group. The values on the bars represented as percentages correspond to the number of the AEs evaluated over the total number of participants of that age range.

Only the safety group reported nonimmediate AEs. However, immediate AEs, postvaccination AEs of grade 3 or more, any medical consult due to an EA, and serious AEs/AEs of special interest (SAEs/AESI) were recorded for all participants. The rationale for this measure is that, as this is a multicenter study performed in several countries, the safety data measured in the subgroups of each center are representative of the entire population studied. The most frequent local nonimmediate AE was pain, observed in around 15% of subjects in the 3- to 11-year age group after the first dose and 8% after the second dose (*P* = 0.003). In the 12- to 17-year age group, pain was reported in around 25% of subjects after each dose, which was significantly higher than the frequency reported in the 3- to 11-year age group after the second dose (*P* = 0.006). The remaining local AEs were reported in less than 5% of subjects in the 3- to 11-year age group and less than 10% in the 12- to 17-year age group. The nonimmediate AEs lasted between 0 and 2 days. For systemic AEs, the overall duration was 0 to 2 days. However, some AEs, such as “skin or mucosa abnormality,” maintained durations of 4.3 days (dose 1) and 5 days (dose 2) for the 3- to 11-year-old group. The duration of “nausea” in subjects 12 to 17 years old after dose 1 was 10.7 days. Finally, in the “other” category, the duration of AEs was 2.3 to 4.3 days for both groups evaluated after vaccination (Fig. 2). These percentages represent solicited AEs within the first week. Nonsolicited AEs are reported up to 28 days after each dose and SAE/AESI throughout the study.

Systemic AEs were reported at a frequency of less than 10% each (Fig. 2). The most frequent systemic AEs were fever for children 3 to 11 years old and headache and fatigue for adolescents. Comparison by age and dose showed a significantly higher frequency of headache in adolescents than in children after both doses (*P* = 0.002 and 0.003) and a higher frequency of fatigue in the 3- to 11-year age group after the first dose than after the second dose (*P* = 0.012). There were 8 allergic reactions only in the 3- to 11-year-old group, and only 2 of them were considered probably related to vaccination. However, none of the volunteers suffered anaphylaxis (Table S2). The severities of systemic AEs were grade 1 in 62 to 79% of participants and grade 3 in only 1.7 to 2.7%. There was no grade 4 AE. All previous medical histories were recorded, but specific symptoms such as headaches were not. Symptoms of myocarditis, such as fatigue, are specifically asked about on the reactogenic daily card, but any other symptoms and hospitalizations should also be reported. In this way, we would be able to detect any picture suggestive of myocarditis.

10.1128/mbio.01311-22.8 TABLE S2 Description of allergic reactions following vaccination in the 3- to 11-year age group. Download Table S2, PDF file, 0.02 MB. Copyright © 2022 Soto et al. 2022 Soto et al. https://creativecommons.org/licenses/by/4.0/ This content is distributed under the terms of the Creative Commons Attribution 4.0 International license.

There was just one nonrelated SAE reported in the period (a 3-year-old participant hospitalized for 24 h due to influenza A infection).

### Two doses of CoronaVac increase antibody titers with neutralizing capacity in children and adolescents.

**FIG 3:**
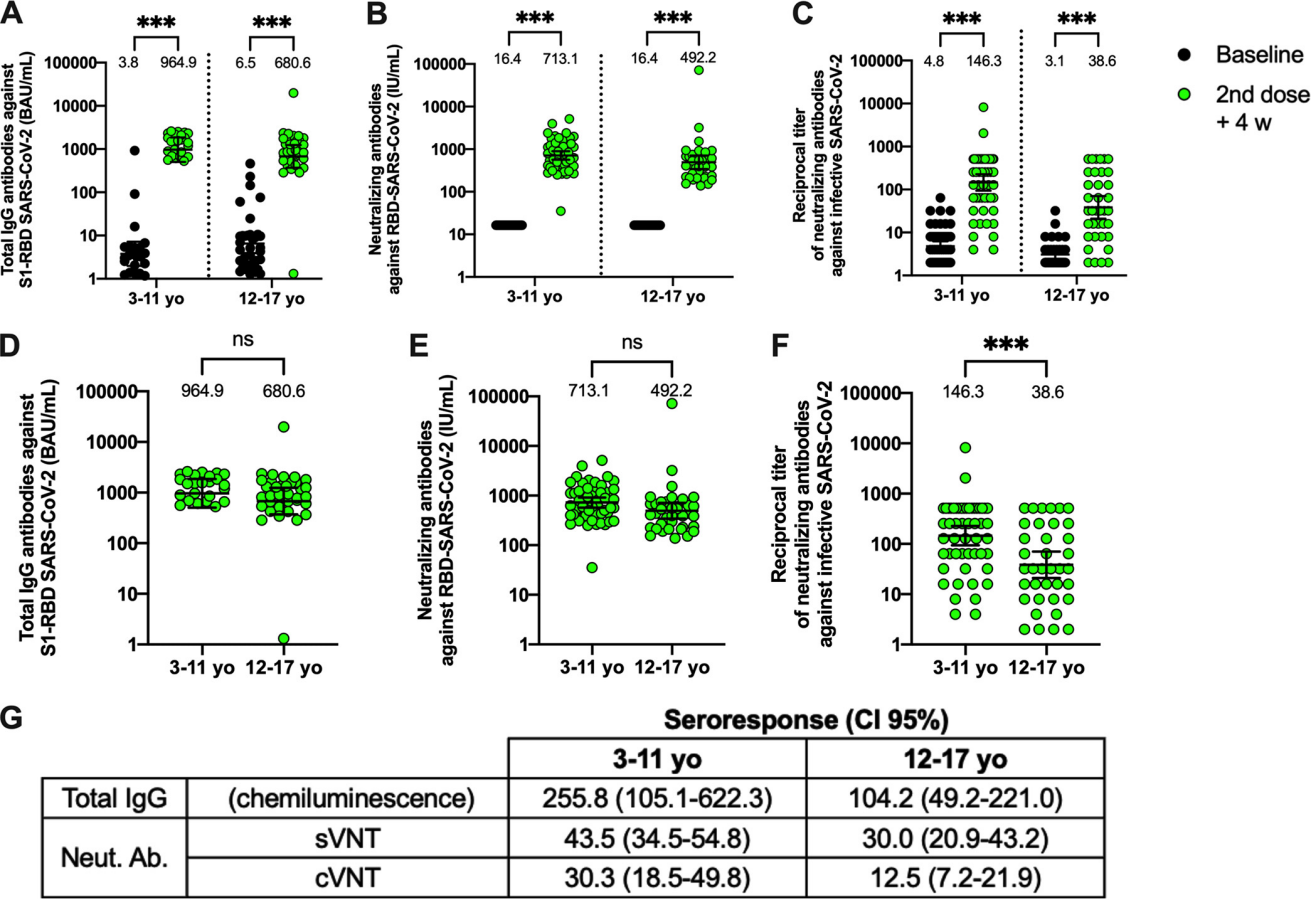
CoronaVac immunization induces anti-S1 RBD antibodies with neutralizing capacities in children and adolescents after two vaccine doses. (A) Total IgG anti-S1 RBD antibodies were detected by chemiluminescence in the plasma of participants immunized with CoronaVac. Results were obtained from 25 participants in the 3- to 11-year age group and 36 participants in the 12- to 17-year age group. (B) Neutralizing antibodies were detected in plasma using a surrogate viral neutralization test (sVNT), which quantifies the interaction between S1 RBD and hACE2 on ELISA plates. Results were obtained from 55 participants in the 3- to 11-year age group and 36 participants in the 12- to 17-year age group. Data are represented as WHO international units per milliliter. (C) Neutralizing antibody titers in plasma were evaluated using a conventional virus neutralizing test (cVNT) in 27 children in the 3- to 11-year age group and 34 participants in the 12- to 17-year age group. A comparison between the 3- to 11-year age group and the 12- to 17-year age group was performed for total IgG anti-S1 RBD antibodies (D), sVNT (E), and cVNT (F). Seroresponse was calculated as the ratio of the geometric means of the 2nd dose plus 4 weeks to baseline (G). The numbers above each set of individual data points show the geometric mean, and the error bars indicate the 95% confidence interval (CI). A two-way ANOVA was followed by Šídák's multiple-comparison test (A to C) and unpaired *t* test (D to F) on the log_10_-transformed data to evaluate differences. ***, *P* < 0.001; ns, nonsignificant.

Ninety-two participants from the immunogenicity branch, who received two doses of CoronaVac, were included in this study (Fig. S1). Clinical data from these patients are described in Table S3. Samples from the placebo group were not analyzed as they did not receive two doses prior to starting the data blind. The samples analyzed were obtained before vaccination (baseline) and 4 weeks after the second dose. IgG against the S1 receptor-binding domain (RBD) of SARS-CoV-2 was significantly increased in plasma samples obtained 4 weeks after the second dose of CoronaVac compared to the baseline sample in both age groups (Fig. 3A). Accordingly, we detected significant neutralizing capacity by a surrogate virus neutralization test (sVNT) in plasma in both age groups 4 weeks after the second dose (Fig. 3B), which is in line with previous reports in adult cohorts (2). Similarly, when analyzing neutralization with a conventional VNT (cVNT), we observed a significant increase in both age groups (Fig. 3C and Table S4A). In addition, the seropositivity reached 96% for the 3- to 11-year age group and 94.5% for the 12- to 17-year age group. However, 100% of seropositivity was found for the samples analyzed by sVNTs 4 weeks after the second dose in both groups, while it reached 100% for the 3- to 11-year age group and 88.2% for the 12- to 17-year age group for cVNT (Table S4A). When comparing the humoral immune responses of both age groups 4 weeks after the second dose, significant differences were found only for cVNT assays (Fig. 3D to F). These results were corroborated with the seroresponse (geometric mean 2nd dose plus 4 weeks versus the baseline) found, which was higher for the 3- to 11-year age group than the 12- to 17-year age group (Fig. 3G).

10.1128/mbio.01311-22.2 FIG S1 Sample size included in immunogenicity assays. From the total number of participants enrolled in one clinical center (CL01, Marcoleta), 115 received two doses of CoronaVac in a 4-week interval (0- to 28-day schedule of vaccination). Ninety-two of them were included in this study. Sixty-one participants were selected to analyze total antibodies by chemoelectroluminescence, 92 were tested for neutralizing antibodies by sVNT, 89 were tested for neutralizing antibodies by cVNT, and 88 were selected to analyze neutralizing antibodies by pVNT. Seventy-four participants were selected to analyze cellular immunity by flow cytometry, 32 were analyzed for cellular responses against variants of concern by flow cytometry, and 46 were analyzed by Luminex. Download FIG S1, TIF file, 0.4 MB. Copyright © 2022 Soto et al. 2022 Soto et al. https://creativecommons.org/licenses/by/4.0/ This content is distributed under the terms of the Creative Commons Attribution 4.0 International license.

10.1128/mbio.01311-22.9 TABLE S3 Baseline characteristics of 92 participants in immunogenicity group. Download Table S3, PDF file, 0.01 MB. Copyright © 2022 Soto et al. 2022 Soto et al. https://creativecommons.org/licenses/by/4.0/ This content is distributed under the terms of the Creative Commons Attribution 4.0 International license.

10.1128/mbio.01311-22.10 TABLE S4 (A) Seropositivity rates, geometric mean units (GMU), or geometric mean titers (GMT) of circulating antibodies against SARS-CoV-2; (B) seropositivity rates and GMT of circulating antibodies against variants of concern of SARS-CoV-2. Download Table S4, PDF file, 0.02 MB. Copyright © 2022 Soto et al. 2022 Soto et al. https://creativecommons.org/licenses/by/4.0/ This content is distributed under the terms of the Creative Commons Attribution 4.0 International license.

Of note, volunteers enrolled in the study were considered seronegative subjects determined by a rapid IgG/IgM antibody test, excluding those seropositive for further immunogenicity assays. The results show that some volunteers presented total and neutralizing anti-SARS-CoV-2 antibodies before vaccination (Fig. 3A and C). These results could be explained by volunteers undergoing asymptomatic infections, whose titers were not detected by the rapid diagnostic tests carried out during the enrollment. These differences in the inclusion criteria could be associated with the increased sensitivity of the techniques used in these trials compared to the rapid test used at enrollment.

To rule out any possible infection that occurred during the evaluation period, volunteers who had any type of symptoms related to a SARS-CoV-2 infection underwent a diagnostic test using reverse transcription-quantitative PCR (RT-qPCR).

### CoronaVac induces a robust activation and memory population of CD4^+^ T cells in children and adolescents.

**FIG 4:**
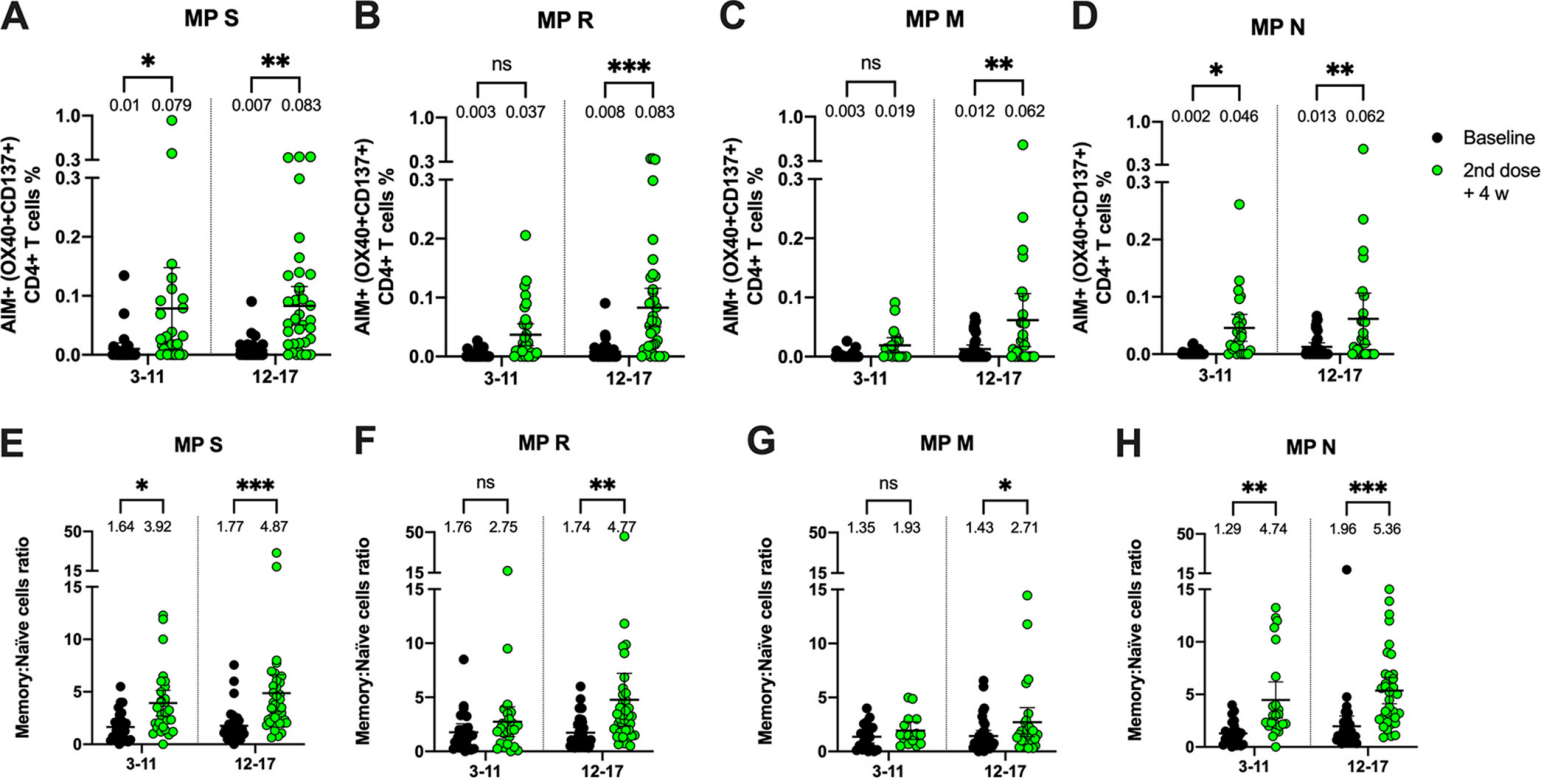
Changes in activation-induced marker (AIM) expression in CD4^+^ T cells and memory AIM^+^ CD4^+^ T cells specific for SARS-CoV-2 after two doses of CoronaVac in children and adolescents. AIM^+^ CD4^+^ T cells were quantified in peripheral blood mononuclear cells (PBMCs) from participants that received two doses of CoronaVac by flow cytometry upon stimulation with megapools (MPs) of peptides derived from SARS-CoV-2 proteins. The percentages of activated AIM^+^ CD4^+^ T cells (OX40^+^, CD137^+^) were determined upon stimulation for 24 h with MPs S, R, M, and N in baseline samples and with samples obtained 4 weeks after the second dose. Data from flow cytometry were normalized against dimethyl sulfoxide (DMSO). Percentages of AIM^+^ CD4^+^ T cells against MPs S, R, M, and N were obtained from a total of 51 participants in the 3- to 11-year age group and 38 participants in the 12- to 17-year age group (A). Memory AIM^+^ CD4^+^ T cells were quantified in PBMCs from participants that received two doses of CoronaVac upon stimulation with MPs of peptides derived from SARS-CoV-2 proteins. The percentages of memory-activated AIM^+^ CD4^+^ T cells (OX40^+^, CD137^+^, CD45RA−, CCR7^+/−^) were determined upon stimulation for 24 h with MPs S, R, M, and N in samples obtained at baseline and 4 weeks after the second dose. Data from flow cytometry were normalized against DMSO. Memory AIM^+^ CD4^+^ T cells against the MPs S, R, M, and N were obtained from a total of 51 participants in the 3- to 11-year age group and 38 participants in the 12- to 17-year age group (B). The numbers above each individual point data set represent the mean, and the error bars are the 95% CI. A two-way ANOVA followed by Šídák's multiple-comparison test was used to assess differences in panels A and B. *, *P* < 0.05; **, *P* < 0.01; ***, *P* < 0.001; n.s., nonsignificant.

We also analyzed the cellular immune responses following two doses of CoronaVac in children and adolescents, which, to our knowledge, has not been reported in other studies with CoronaVac or mRNA vaccines against SARS-CoV-2. Compared to the baseline samples, we observed a significant increase in CD4^+^ T cell activation 4 weeks after the second dose of CoronaVac upon stimulation with four megapools (MPs) comprising peptides from the S, R (all but S), M, and N viral proteins (Fig. S2A and B). A significant increase in the activation of CD4^+^ T cells was found in the 12- to 17-year age group for all the MPs evaluated. In contrast, the increase in the activation of CD4^+^ T cells for the 3- to 11-year age group was statistically significant for the S and N stimuli only (Fig. 4A). Additionally, the induction of memory markers in activated CD4^+^ T cells induced 4 weeks after the second dose was compared to the baseline samples (Fig. S2C and D). An increase in the ratio of memory cells with respect to the baseline samples was observed in the 12- to 17-year age group in the presence of all stimuli (Fig. 4B). For the 3- to 11-year age group, an increase in the ratio of memory cells with respect to the baseline samples was observed only in the presence of the S and N stimuli (Fig. 4B).

10.1128/mbio.01311-22.3 FIG S2 Representative plots in activation-induced marker (AIM) expression in CD4^+^ T cells specific for SARS-CoV-2 after two doses of CoronaVac in children and adolescents. AIM^+^ CD4^+^ T cells were quantified in peripheral blood mononuclear cells of participants that received two doses of CoronaVac by flow cytometry, upon stimulation with megapools (MPs) of peptides derived from SARS-CoV-2 proteins. The percentages of activated AIM^+^ CD4^+^ T cells (OX40^+^, CD137^+^) were determined upon stimulation for 24 h with MPs S, R, M, and N in baseline samples and samples obtained 4 weeks after the second dose. Representative flow cytometry plots for participants in the 3-to 11-year age group (A) and 12- to 17-year age group (B) are shown. The percentages of memory AIM^+^ CD4^+^ T cells (OX40^+^, CD137^+^, CD45RA^−^, CCR7^+/−^) were determined upon stimulation for 24 h with MPs S, R, M, and N in baseline samples and samples obtained 4 weeks after the second dose. Representative flow cytometry plots for participants in the 3- to 11-year age group (C) and in the 12- to 17-year age group (D) are shown. The different representative plots are from different volunteers. Download FIG S2, TIF file, 1.3 MB. Copyright © 2022 Soto et al. 2022 Soto et al. https://creativecommons.org/licenses/by/4.0/ This content is distributed under the terms of the Creative Commons Attribution 4.0 International license.

Moreover, secretion of the cytokines interleukin-2 (IL-2) and gamma interferon (IFN-γ) by peripheral blood mononuclear cells (PBMCs) stimulated with MPs of peptides was evaluated using Luminex. We observed a significant increase in IL-2 secretion in response to the S and R MPs and M and N MPs in the 12- to 17-year age group (Fig. S3A to D). In the case of the 3- to 11-year age group, we observed a significant increase in response to the S, M, and N MPs. In contrast, we did not observe a significant increase in IFN-γ release after the MP stimulation for participants of ages 12 to 17 years (Fig. S3E to H). However, the production of IFN-γ in response to the S, M, and N MPs showed a significant increase in the 3- to 11-year age group (Fig. S3E to H). No differences were found in IL-4 levels after vaccination, measured by Luminex technology (data not shown). Levels of IFN-γ and IL-4 were also evaluated by enzyme-linked immunosorbent spot (ELISPOT), but we did not observe significant differences in any of these cytokines upon stimulation with SARS-CoV-2 MPs (Fig. S4A to H). We were not able to evaluate IL-2 by ELISPOT due to the limited number of cells obtained from each volunteer.

10.1128/mbio.01311-22.4 FIG S3 Changes in IL-2 and IFN-γ secretion by PBMCs stimulated with SARS-CoV-2 megapools of peptides after two doses of CoronaVac in children and adolescents. IL-2 and IFN-γ secretion was quantified in supernatants of peripheral blood mononuclear cells of participants that received two doses of CoronaVac, upon stimulation with megapools of peptides derived from SARS-CoV-2 proteins for 18 h by Luminex. Levels of IL-2 secretion against the MPs S (A), R (B), M (C), and N (D) and IFN-γ secretion against the MPs S (E), R (F), M (G), and N (H) are shown from a total of 23 participants 3 to 11 years old and 23 participants 12 to 17 years old. A two-way ANOVA was used to compare the level of cytokines 4 weeks after the second dose against the baseline sample. *, *P* < 0.05; **, *P* < 0.01; ***, *P* < 0.001; n.s., nonsignificant. Download FIG S3, TIF file, 0.4 MB. Copyright © 2022 Soto et al. 2022 Soto et al. https://creativecommons.org/licenses/by/4.0/ This content is distributed under the terms of the Creative Commons Attribution 4.0 International license.

10.1128/mbio.01311-22.5 FIG S4 Expression levels of IL-4 and IFN-γ from specific T cells against MPs of SARS-CoV-2 after two doses of CoronaVac in children and adolescents. IL-4 and IFN-γ secretion from peripheral blood mononuclear cells of volunteers that received two doses of CoronaVac, upon stimulation with megapools of peptides derived from SARS-CoV-2 proteins for 48 h by ELISPOT. Spot-forming cells (SFCs) from IFN-γ secretion against the MPs S (A), R (B), M (C), and N (D) and IL-4 secretion against the MPs S (E), R (F), M (G), and N (H) are shown from a total of 23 volunteers 3 to 11 years old and 23 volunteers aged 12 to 17 years old. A two-way ANOVA was used to compare the level of cytokines 4 weeks after the second dose against the preimmune sample. ns, no significant. Download FIG S4, TIF file, 0.4 MB. Copyright © 2022 Soto et al. 2022 Soto et al. https://creativecommons.org/licenses/by/4.0/ This content is distributed under the terms of the Creative Commons Attribution 4.0 International license.

We did not observe an increase in CD8^+^ activation-induced marker-positive (AIM^+^) T cells with MP CD8A and CD8B in any age group following the second dose of CoronaVac compared to the baseline sample (data not shown). However, we found significant differences for memory CD8^+^ AIM^+^ T cell analyses (Fig. S5). Representative flow cytometry plots of memory CD8^+^ AIM^+^ T cells for participants in the 3- to 11-year age group (Fig. S5A) and 12- to 17-year age group (Fig. S5B) are shown. A significant increase in memory CD8^+^ AIM^+^ T cells after vaccination with CoronaVac was observed only upon stimulation with MP CD8A in the 3- to 11-year age group (Fig. S5C). Both groups found no differences upon stimulation with MP CD8B (Fig. S5D).

10.1128/mbio.01311-22.6 FIG S5 Changes in memory AIM^+^ CD8^+^ T cells specific for SARS-CoV-2 after two doses of CoronaVac in children and adolescents. Memory AIM^+^ CD8^+^ T cells were quantified in peripheral blood mononuclear cells of volunteers that received two doses of CoronaVac, upon stimulation with megapools of peptides derived from SARS-CoV-2 proteins. The percentages of memory activated AIM^+^ CD8^+^ T cells (CD69^+^, CD137^+^, CD45RA^−^, CCR7^+/−^) were determined upon stimulation for 24 h with MPs S, R, M, and N in samples obtained at the preimmune stage and 4 weeks after the second dose. Data from flow cytometry were normalized against DMSO and analyzed separately by a Wilcoxon test against the baseline. Representative flow cytometry plots for volunteers (A) 3 to 11 years old and (B) 12 to 17 years old are shown. Memory AIM^+^ CD8^+^ T cells against the MPs CD8A (C) and CD8B (D) were obtained from a total of 30 volunteers 3 to 11 years old and 30 volunteers 12 to 17 years old. A two-way ANOVA was used to compare the percentage of memory AIM^+^ CD8^+^ T cells 4 weeks after the second dose against the baseline sample in both age groups. *, *P* < 0.05; n.s., nonsignificant. The different representative plots are from different volunteers. Download FIG S5, TIF file, 0.8 MB. Copyright © 2022 Soto et al. 2022 Soto et al. https://creativecommons.org/licenses/by/4.0/ This content is distributed under the terms of the Creative Commons Attribution 4.0 International license.

### Neutralizing antibodies and specific T cells induced by two doses of CoronaVac in children and adolescents recognize Delta and Omicron variants of SARS-CoV-2.

**FIG 5:**
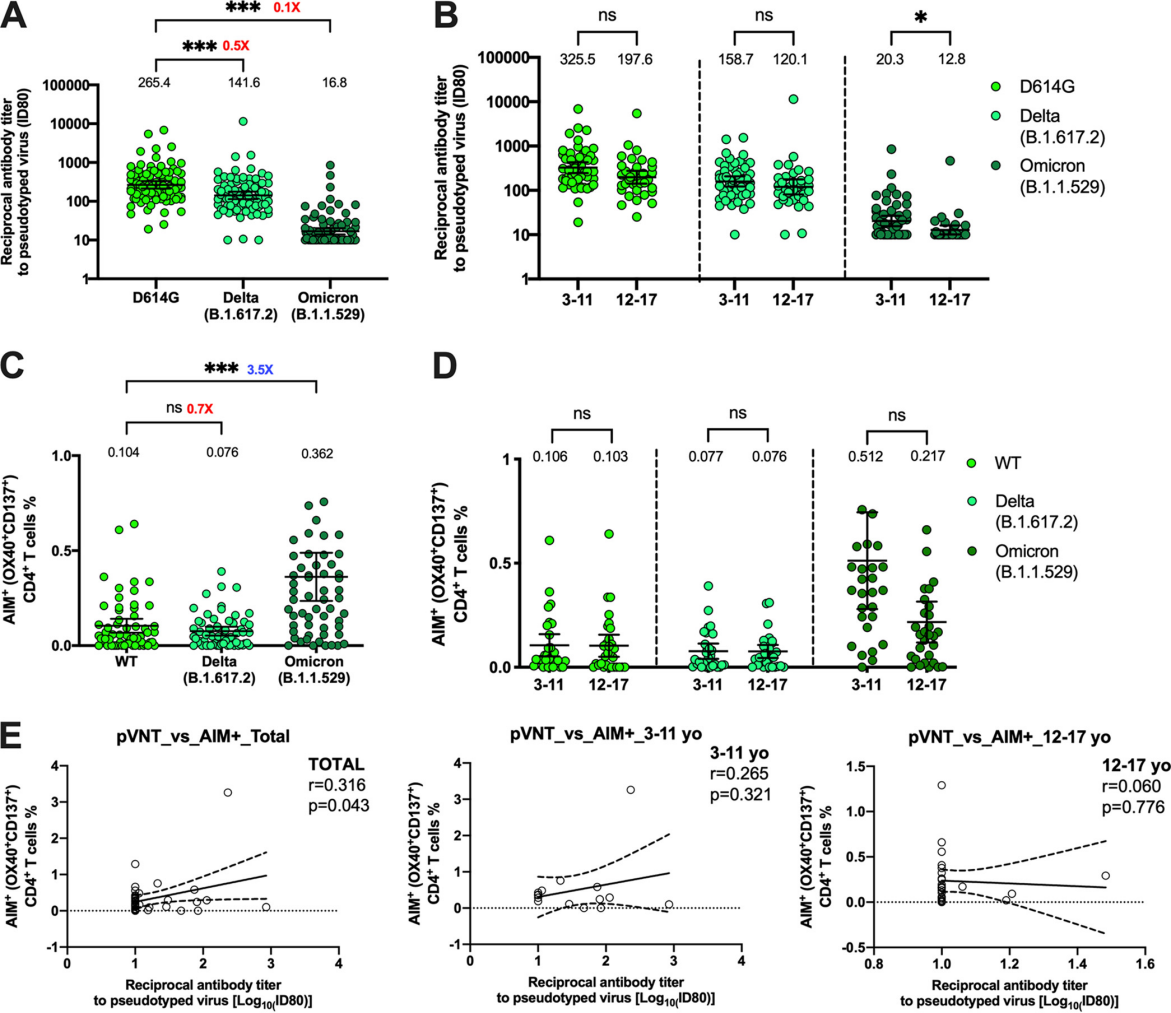
Quantification of circulating neutralizing antibodies and AIM^+^ CD4^+^ T cells against SARS-CoV-2 variants Delta and Omicron in participants that received two doses of CoronaVac. (A) Neutralizing antibodies were detected in the plasma of 88 participants 4 weeks after the second dose of CoronaVac, using a pseudotyped virus neutralization test (pVNT). Data are expressed as the reciprocal of the highest dilution preventing 80% of the infection (ID_80_). Numbers above the bars show the GMT, and the error bars indicate the 95% CI. (B) Neutralizing antibody levels between 52 participants in the 3- to 11-year age group and 36 participants in the 12- to 17-year age group against D614G, Delta, and Omicron variants are shown. (C and D) AIM^+^ CD4^+^ T cells against the variants Delta and Omicron were measured by flow cytometry. (C) Results were obtained from a total of 59 participants. (D) Results shown by age group (29 participants from the 3- to 11-year age group and 30 participants from the 12- to 17-year age group). The numbers in red (decrease) or blue (increase) next to the statistics show the fold change of the response against the variant relative to the WT or D614G strain. (E) The correlations between neutralizing antibody titers and AIM^+^ percentages against the Omicron variant were evaluated. The Pearson correlation coefficient (*r*) and the *P* value are indicated in each scatterplot. A one-way ANOVA followed by Dunnett’s multiple-comparison test (A and C) and two-way ANOVA followed by Šídák’s multiple-comparison test (B and D) were performed to assess differences. *, *P* < 0.05; ***, *P* < 0.001; n.s., nonsignificant.

To assess whether CoronaVac induces immune responses against SARS-CoV-2 VOC, we evaluated by a pseudotyped virus neutralization assay (pVNT) the neutralizing antibody levels against VOC Delta and Omicron comparing them to the D614G strain (which was used as a control as it has a Spike RBD identical to the ancestral strain but contains a mutation outside the RBD, which is also contained in most of the VOC) (Fig. 5). A 1.9-fold reduction relative to strain D614G was found in neutralization against Delta, while a 15.8-fold reduction was observed against Omicron (Fig. 5A). The percentages of seropositivity showed an important reduction for the Omicron variant (Table S4B). When we compared the responses between both age groups, we did not find significant differences for the D614G and Delta variants. However, a significantly higher level of neutralizing antibodies against Omicron was observed in the 3- to 11-year age group (Fig. 5B). A mild but insignificant reduction of AIM^+^ T cells against MP-S of the Delta variant (1.4-fold reduction) and a significant increase against MP S of the Omicron variant (3.5-fold increase) were observed, compared to the response obtained for MP S of the wild-type (WT) strain (Fig. 5C). This T cell response was equivalent in both age groups (Fig. 5D). While Fig. 5B and D suggest a possible correlation between the humoral and cellular responses against Omicron in the 3- to 11-year age group, it was not possible to find a positive correlation between antibody titers against Omicron and CD4^+^ T cell response (Fig. 5E).

## DISCUSSION

Multiple vaccines against SARS-CoV-2 have been evaluated in clinical trials. However, trials addressing the immune response in the pediatric population are scarce. Here, we report that CoronaVac has a good safety profile in children and adolescents in Chile, comparable to what was reported by Han et al. (6), with pain as the main AE in both age groups but statistically more frequently reported in adolescents than in children. Most AEs were mild or moderate, and no SAEs related to the vaccine were reported. This is in line with previous reports in adults vaccinated with CoronaVac in Chile and other countries, which have mainly reported mild or moderate AEs in participants 18 to 59 years old and older than 60 years (2, 4, 5). Of note, we acknowledge that the safety follow-up reported here is relatively short term. Indeed, for rare SAEs such as myocarditis, the sample size may be small and the observation time too short to detect any cases.

We also show that two doses of CoronaVac, administered in a 4-week interval, stimulate the induction of both total and neutralizing antibodies in participants 3 to 17 years old 4 weeks after the second dose. This is the first report of total IgG antibodies anti-S1 RBD expressed as WHO international units in children and adolescents vaccinated with CoronaVac, allowing their comparison to other vaccine platforms. However, clinical trials in children and adolescents have only reported neutralizing antibodies against SARS-CoV-2 (7, 8).

Our results suggest that CoronaVac promotes CD4^+^ T cell responses against SARS-CoV-2, which can be protective against infection and/or severe disease. Here, we report a significant increase in CD4^+^ AIM^+^ T cells in response to MPs but no differences in CD8^+^ AIM^+^ T cells, in line with the results previously observed in vaccinated adults, which indicate that CoronaVac is better at inducing CD4^+^ T cell responses. We did not observe significant differences between age groups in CD4^+^ AIM^+^ T cells, suggesting that children and adolescents can activate CD4^+^ T cell responses against SARS-CoV-2 following vaccination. The 12- to 17-year age group exhibit increased CD4^+^ T cell activation against all the SARS-CoV-2 MP evaluated, which indicate that the vaccine stimulates cellular immunity not only against the Spike protein but also against the M and N proteins, consistent with the fact that this is an inactivated virus vaccine containing the major structural proteins. However, the 3- to 11-year age group did not exhibit increased CD4^+^ T cell activation against the M protein. Consistent with this, we show a significant increase in IL-2 secretion in response to S and N MPs in both age groups, whereas we detected a significant increase in response to the M MPs only in subjects 12 to 17 years old. As previously reported in adults vaccinated with CoronaVac (2), we did not observe an increase in IL-4 production in stimulated cells, indicating that CoronaVac stimulates a Th1 profile in vaccinated children and adolescents. Similarly, we observed a significant increase in IFN-γ production using Luminex technology in the 3- to 11-year age group upon stimulation with the MP-S but not in the other age group and in the presence of other MPs. However, this significant difference may be attributed to the high levels of IFN-γ in some subjects but is not necessarily representative of the whole group. In line with this, we did not observe significant differences in this cytokine using ELISPOT. Furthermore, we observed an increase in the frequency of memory CD4^+^ AIM^+^ T cells in response to SARS-CoV-2 MP-S and slightly higher induction of memory T cells in subjects 12 to 17 years old than in subjects 3 to 11 years old. This increased ratio of memory CD4^+^ T cells suggests that the vaccine may induce durable CD4^+^ T cell responses. However, evaluation of these responses at later time points following the second dose are required to confirm this idea. These results agree with reports showing that memory T cell responses against SARS-CoV-2 structural proteins increase with age (9).

Moreover, we evaluated the neutralization using a pseudotyped virus against variants Delta and Omicron compared to the ancestral strain D614G and found decreased antibody neutralization capacity against these variants. While we observed high seropositivity against D614G (100%), lower seropositivity against the variant Omicron was found (45.5%) (Table S4B) in line with previous reports indicating lower protection against VOC in adult cohorts after two doses of CoronaVac (10–12). However, a booster dose of CoronaVac has been shown to increase virus neutralization of the VOC Gamma and Delta (13). Thus, a booster dose of CoronaVac may be required to increase virus neutralization of circulating SARS-CoV-2 variants in children and adolescents. This is consistent with our studies and another trial conducted in Israel (14).

On the other hand, we observed that both age groups elicited CD4^+^ AIM^+^ T cells in response to MPs from the variants Delta and Omicron. We did not observe significant differences in CD4^+^ AIM^+^ T cells against the Delta variant compared to the ancestral strain, but surprisingly, an increase against the Omicron variant was observed. Several studies in vaccinated adults have shown that CD4^+^ T cell responses against VOC are conserved, and cross-reactive T cells against the Omicron variant have been reported (15, 16). However, it is unclear why this pediatric population exhibits increased CD4^+^ AIM^+^ T cells against the Omicron variant, and further research is required to understand these results. In this sense, a study in volunteers 12 to 16 years old vaccinated with two doses of ChAdOx1 nCoV-19 or mRNA vaccines showed an effect on the cellular response against the Omicron variant similar to that reported by us, suggesting that the cellular response is more stable against other SARS-CoV-2 variants than the humoral response.

One of the main limitations of this study is the impossibility of repeating a large part of the assays, given that the volumes of blood extracted from the volunteers were very small, especially for the younger subjects. Since, in this study, the monitoring of possible SARS-CoV-2 infections by RT-qPCR was performed for symptomatic cases only, we cannot rule to the presence of asymptomatic cases during the study. Despite this, a study evaluating long-lived immunity observed that seronegative participants who received two doses of BNT162b2 presented a response similar to the reported in this investigation against variants such as Omicron (17), suggesting a low, if any, the contribution of asymptomatic cases to our results. These results indicate that CoronaVac is safe in children and adolescents and induces both humoral and cellular responses able to recognize the VOC Delta and Omicron.

Finally, CoronaVac shows promising results in the induction of the immune response against SARS-CoV-2 and the variants of interest. However, to date, there is limited information regarding the long-term immune response in the pediatric population, whose information is essential for the implementation of vaccination schedules. In this sense, a study comparing adult volunteers vaccinated with two doses of CoronaVac and receiving a booster with CoronaVac, BNT162b2, and ChAdOx1 after 6 to 8 months showed that the best immune response is induced when the booster is a vaccine different than CoronaVac. However, no differences were found in the three groups evaluated when the humoral response was compared 30 or 100 days after the booster (18), suggesting that a booster dose with either CoronaVac or other vaccine platforms may be beneficial in pediatric individuals. Further studies are required to assess the immune responses using a heterologous or homologous vaccination schedule.

## MATERIALS AND METHODS

### Study design.

This study is a global multicenter, randomized, double-blind, and placebo-controlled phase 3 clinical trial that aims to assess the safety, efficacy, and immunogenicity of CoronaVac among children aged 6 months to 17 years. The administration of placebo and vaccine was in a ratio of 1:1. Four countries participated in this study, including South Africa, Malaysia, Philippines, and Chile (ClinicalTrials registration no. NCT04992260). This report will focus only on the study performed in Chile for participants that received CoronaVac. In Chile, this trial has been conducted at 11 different sites, eight in the center of the country (seven in Santiago and one in Valparaiso), two in the South (Puerto Montt and Valdivia), and one in the North (Antofagasta) of Chile. The study protocol was conducted according to the current *Tripartite Guidelines for Good Clinical Practices*, the Declaration of Helsinki (19), and local regulations. This trial was approved by each Institutional Ethical Committee (ID 210616012) and the Chilean Public Health Institute (ISP Chile, no. 20674/21). Written informed consent was obtained from the parent(s) or legal representative(s) of the child before enrollment. Assent was obtained from individuals from 7 years old. Participants did not receive any payment for their participation. The study included children and adolescents 3 to 17 years old who were inoculated with two doses of 3 μg (600 SU) of CoronaVac in a 4-week interval (0- to 28-day schedule) (Fig. 1A). Before the volunteers were vaccinated, a negative rapid antigen test result was required. Exclusion criteria included, among others, a history of confirmed symptomatic SARS-CoV-2 infection, pregnancy, allergy to vaccine components, and immunocompromised condition. Well-controlled medical conditions were allowed. A complete list of inclusion/exclusion criteria is provided in the supplemental material.

### Study population and outcomes.

Participants were assigned to the age groups 3 to 5 (children), 6 to 11 (children), and 12 to 17 (adolescents) years and immunogenicity subgroup, safety subgroup, or nonsubgroup. The nonsubgroup, which included only children 3 to 5 years old with vaccine and placebo, did not have a daily follow-up after the vaccination and only had a follow-up if the members of the group reported serious adverse events (SAEs) and adverse events of special interest (AESI). The initial protocol had a placebo for the 3- to 5-year-old arm and was a double-blind study, but this was modified to an open-label study due to the approval of the emergency vaccination to the general population in Chile. Therefore, the nonsubgroup (3 to 5 years old) was redistributed to the immunogenicity and safety arms. After the unblinding, 158 children who received a placebo were reenrolled to be immunized with CoronaVac. For the present study, we combined the children in the 3- to 11-year age group (Fig. 1B). Besides, only volunteers with two doses of CoronaVac until 31st December 2021 were included in these analyses. Participants not considered in this article received their second dose after 31st December 2021. The safety group includes registration of every local and systmic nonimmediate adverse events (AEs) 7 days after vaccination and any other AE until 28 days after each dose. For all participants, immediate AEs (30 min postvaccination) and SAEs as well as AESI were recorded. The safety studies considered the subjects enrolled until 31st December 2021, enrolled from the 11 centers in Chile. The safety data on the other countries participating in the study were not included since the global safety data set of the entire study is only managed by the sponsoring company, Sinovac, and until the closing date of this article, there was no information on all of the countries.

The study aims were to evaluate the immunogenicity of CoronaVac in a subgroup of participants 4 weeks after 2 doses and the frequency of solicited immediate (first 30 min postdose) and nonimmediate adverse events (AEs) that occur during 7 days after each dose, stratified by age group (3 to 11 and 12 to 17 years old). The frequency of SAEs/AESI and any other AEs represents events that occur 28 days after each dose, and the frequency of any SAE/AESI represents events that occur 12 months after the second dose.

### Sample collection.

Subjects enrolled in one specific clinical center (CL01, Marcoleta) were assigned to the immunogenicity branch, which considered 148 volunteers. Blood samples were obtained in heparinized tubes before administration of the first dose (baseline) and 4 weeks after the second dose as described in Fig. S1 in the supplemental material. Samples were used to obtain plasma and peripheral blood mononuclear cells (PBMCs) and stored at −80°C (plasma) and –170°C (PBMCs) until humoral and cellular immunity analyses were performed. The sample size included in each experimental analysis is described in Fig. S1.

### Procedures.

IgG anti-S1 RBDs of SARS-CoV-2 were tested using ADVIA Centaur XP SARS-CoV-2 IgG (sCOVG) (Siemens) (20, 21), an automated two-step sandwich antibody-binding immunoassay using indirect chemiluminescence. sCOVG was used for quantitative detection expressed in binding antibody units (BAU) per milliliter after interpolating the WHO standard NIBSC code 20/136 calibration (21). The cutoff considered for this methodology was 1.18 U/mL. The presence of circulating antibodies able to block the interaction of the RBD of the S1 subunit of the SARS-CoV-2 Spike protein with the recombinant human angiotensin 2 receptor (hACE2) was evaluated using a surrogate virus neutralization test (sVNT) (Genscript catalog no. L00847-A). A cutoff of >30% was used to define if samples were positive or not for the presence of anti-SARS-CoV2 neutralizing antibodies. Seropositivity designation and transformation to WHO arbitrary units were described previously (13). As previously reported, conventional virus neutralization tests (cVNTs) to detect neutralization against the ancestral SARS-CoV-2 virus were performed. The neutralization titer was calculated considering 100% inhibition of the virus, which was related to an absence of cytopathic effect on Vero E6 cells (2). A pseudotyped virus neutralization test (pVNT) assay was performed to assess neutralization against SARS-CoV-2 VOC, as previously reported (12, 22) (Text S1). To evaluate the cellular immune response, PBMCs of 60 participants were stimulated with six megapools (MPs) derived from the SARS-CoV-2 proteome (23) (Text S1). MPs of peptides from the S protein of SARS-CoV-2 VOC Delta and Omicron were provided by La Jolla Institute for Immunology (16). Positive and negative controls were included in each assay. The numbers of spot-forming cells (SFCs) for IFN-γ and IL-4 were determined by ELISPOT, and the expression of activation-induced marker (AIM^+^) and memory markers by T cells was evaluated by flow cytometry using a LSR Fortessa X-20 flow cytometer (BD Biosciences). Assays were performed according to the manufacturer’s instructions as reported previously (2). Supernatants from PBMCs stimulated for 20 h with SARS-CoV-2 MPs were evaluated using the Luminex technology (R&D Systems, USA) to assess IL-2 and IFN-γ production (Text S1).

10.1128/mbio.01311-22.1 TEXT S1 Supplemental materials and methods. Download Text S1, DOCX file, 0.1 MB. Copyright © 2022 Soto et al. 2022 Soto et al. https://creativecommons.org/licenses/by/4.0/ This content is distributed under the terms of the Creative Commons Attribution 4.0 International license.

The selection of participants for immunogenicity analysis was based on the availability of samples collected from the baseline and 4 weeks post-second dose. In addition, only those subjects who were seronegative at study entry were included.

### Statistical analyses.

Statistical differences in the immunogenicity results were assessed using a two-way ANOVA followed by Šídák's multiple-comparison test to compare the levels of antibodies 4 weeks after the second dose against the baseline levels in the different age groups (total IgG, sVNT, pVNT, and cVNT). Two-way ANOVA followed by Šídák's multiple-comparison tests was also used for cellular immune response to compare the percentages of AIM^+^, memory AIM^+^ CD4^+^ T cells, and cytokine secretion 4 weeks after the second dose against the baseline levels in both age groups. A one-way ANOVA followed by Dunnett's multiple-comparison test was used to evaluate total antibodies or cells from variants of SARS-CoV-2. When indicated, the analyses were performed on the log_10_-transformed data. Regarding clinical data, adverse events among groups were compared with the chi-square test or Fisher exact test. The significance level was set at 0.05 for all the analyses. All data were analyzed with GraphPad Prism 9.4.0 or R version 3.6.1.

### Data availability.

All analyzed and raw data (masked to protect the information of volunteers) are available upon reasonable request to the corresponding authors through email after the publication of this article. A signed data access agreement will be requested to share the data. The study protocol is also available online.

## References

[1] Mallapaty S. 2021. WHO approval of Chinese CoronaVac COVID vaccine will be crucial to curbing pandemic. Nature 594:161–162. doi:10.1038/d41586-021-01497-8. 34089030

[2] Bueno SM, Abarca K, González PA, Gálvez NMS, Soto JA, Duarte LF, Schultz BM, Pacheco GA, González LA, Vázquez Y, Ríos M, Melo-González F, Rivera-Pérez D, Iturriaga C, Urzúa M, Domínguez A, Andrade CA, Berríos-Rojas RV, Canedo-Marroquín G, Covián C, Moreno-Tapia D, Saavedra F, Vallejos OP, Donato P, Espinoza P, Fuentes D, González M, Guzmán P, Muñoz Venturelli P, Pérez CM, Potin M, Rojas Á, Fasce RA, Fernández J, Mora J, Ramírez E, Gaete-Argel A, Oyarzún-Arrau A, Valiente-Echeverría F, Soto-Rifo R, Weiskopf D, Sette A, Zeng G, Meng W, González-Aramundiz JV, Kalergis AM, CoronaVac03CL Study Group. 2022. Safety and immunogenicity of an inactivated severe acute respiratory syndrome coronavirus 2 vaccine in a subgroup of healthy adults in Chile. Clin Infect Dis 75:e792–e804. doi:10.1093/cid/ciab823. 34537835 PMC9402626

[3] Qiang G, Linlin B, Haiyan M, Lin W, Kangwei X, Minnan Y, Yajing L, Ling Z, Nan W, Zhe L, Hong G, Xiaoqin G, Biao K, Yaling H, Jiangning L, Fang C, Deyu J, Yanhui Y, Chengfeng Q, Jing L, Xuejie G, Xiuyu L, Wen S, Dongdong W, Hengming Z, Lang Z, Wei D, Yurong L, Jinxing L, Changgui L, Xiangxi W, Weidong Y, Yanjun Z, Chuan Q. 2020. Development of an inactivated vaccine candidate for SARS-CoV-2. Science 369:77–81. doi:10.1126/science.abc1932. 32376603 PMC7202686

[4] Wu Z, Hu Y, Xu M, Chen Z, Yang W, Jiang Z, Li M, Jin H, Cui G, Chen P, Wang L, Zhao G, Ding Y, Zhao Y, Yin W. 2021. Safety, tolerability, and immunogenicity of an inactivated SARS-CoV-2 vaccine (CoronaVac) in healthy adults aged 60 years and older: a randomised, double-blind, placebo-controlled, phase 1/2 clinical trial. Lancet Infect Dis 21:803–812. doi:10.1016/S1473-3099(20)30987-7. 33548194 PMC7906628

[5] Zhang Y, Zeng G, Pan H, Li C, Hu Y, Chu K, Han W, Chen Z, Tang R, Yin W, Chen X, Hu Y, Liu X, Jiang C, Li J, Yang M, Song Y, Wang X, Gao Q, Zhu F. 2021. Safety, tolerability, and immunogenicity of an inactivated SARS-CoV-2 vaccine in healthy adults aged 18–59 years: a randomised, double-blind, placebo-controlled, phase 1/2 clinical trial. Lancet Infect Dis 21:181–192. doi:10.1016/S1473-3099(20)30843-4. 33217362 PMC7832443

[6] Han B, Song Y, Li C, Yang W, Ma Q, Jiang Z, Li M, Lian X, Jiao W, Wang L, Shu Q, Wu Z, Zhao Y, Li Q, Gao Q. 2021. Safety, tolerability, and immunogenicity of an inactivated SARS-CoV-2 vaccine (CoronaVac) in healthy children and adolescents: a double-blind, randomised, controlled, phase 1/2 clinical trial. Lancet Infect Dis 21:1645–1653. doi:10.1016/S1473-3099(21)00319-4. 34197764 PMC8238449

[7] Ali K, Berman G, Zhou H, Deng W, Faughnan V, Coronado-Voges M, Ding B, Dooley J, Girard B, Hillebrand W, Pajon R, Miller JM, Leav B, McPhee R. 2021. Evaluation of mRNA-1273 SARS-CoV-2 vaccine in adolescents. N Engl J Med 385:2241–2251. doi:10.1056/NEJMoa2109522. 34379915 PMC8385554

[8] Walter EB, Talaat KR, Sabharwal C, Gurtman A, Lockhart S, Paulsen GC, Barnett ED, Muñoz FM, Maldonado Y, Pahud BA, Domachowske JB, Simões EAF, Sarwar UN, Kitchin N, Cunliffe L, Rojo P, Kuchar E, Rämet M, Munjal I, Perez JL, Frenck RW, Lagkadinou E, Swanson KA, Ma H, Xu X, Koury K, Mather S, Belanger TJ, Cooper D, Türeci Ö, Dormitzer PR, Şahin U, Jansen KU, Gruber WC. 2022. Evaluation of the BNT162b2 Covid-19 vaccine in children 5 to 11 years of age. N Engl J Med 386:35–46. doi:10.1056/NEJMoa2116298. 34752019 PMC8609605

[9] Cohen CA, Li APY, Hachim A, Hui DSC, Kwan MYW, Tsang OTY, Chiu SS, Chan WH, Yau YS, Kavian N, Ma FNL, Lau EHY, Cheng SMS, Poon LLM, Peiris M, Valkenburg SA. 2021. SARS-CoV-2 specific T cell responses are lower in children and increase with age and time after infection. Nat Commun 12:4678. doi:10.1038/s41467-021-24938-4. 34326343 PMC8322064

[10] Fernández J, Bruneau N, Fasce R, Martín HS, Balanda M, Bustos P, Ulloa S, Mora J, Ramírez E. 2022. Neutralization of alpha, gamma, and D614G SARS-CoV-2 variants by CoronaVac vaccine-induced antibodies. J Med Virol 94:399–403. doi:10.1002/jmv.27310. 34460119 PMC8662277

[11] Beltrán-Pavez C, Riquelme-Barrios S, Oyarzún-Arrau A, Gaete-Argel A, González-Stegmaier R, Cereceda-Solis K, Aguirre A, Travisany D, Palma-Vejares R, Barriga GP, Gaggero A, Martínez-Valdebenito C, Le Corre N, Ferrés M, Balcells ME, Fernandez J, Ramírez E, Villarroel F, Valiente-Echeverría F, Soto-Rifo R. 2021. Insights into neutralizing antibody responses in individuals exposed to SARS-CoV-2 in Chile. Sci Adv 7:eabe6855. doi:10.1126/sciadv.abe6855. 33579701 PMC7880587

[12] Melo-González F, Soto JA, González LA, Fernández J, Duarte LF, Schultz BM, Gálvez NMS, Pacheco GA, Ríos M, Vázquez Y, Rivera-Pérez D, Moreno-Tapia D, Iturriaga C, Vallejos OP, Berríos-Rojas RV, Hoppe-Elsholz G, Urzúa M, Bruneau N, Fasce RA, Mora J, Grifoni A, Sette A, Weiskopf D, Zeng G, Meng W, González-Aramundiz JV, González PA, Abarca K, Ramírez E, Kalergis AM, Bueno SM. 2021. Recognition of variants of concern by antibodies and T cells induced by a SARS-CoV-2 inactivated vaccine. Front Immunol 12:747830. doi:10.3389/fimmu.2021.747830. 34858404 PMC8630786

[13] Schultz BM, Melo-González F, Duarte LF, Gálvez NMS, Pacheco GA, Soto JA, Berríos-Rojas RV, González LA, Moreno-Tapia D, Rivera-Pérez D, Ríos M, Vázquez Y, Hoppe-Elsholz G, Andrade-Parra CA, Vallejos OP, Piña-Iturbe A, Iturriaga C, Urzua M, Navarrete MS, Rojas Á, Fasce R, Fernández J, Mora J, Ramírez E, Gaete-Argel A, Acevedo ML, Valiente-Echeverría F, Soto-Rifo R, Weiskopf D, Grifoni A, Sette A, Zeng G, Meng W, CoronaVac03CL Study Group, González-Aramundiz JV, González PA, Abarca K, Kalergis AM, Bueno SM. 2022. A booster dose of coronavac increases neutralizing antibodies and t cells that recognize delta and omicron variants of concern. mBio 13:e01423-22. doi:10.1128/mbio.01423-22. 35946814 PMC9426482

[14] Bar-On YM, Goldberg Y, Mandel M, Bodenheimer O, Freedman L, Kalkstein N, Mizrahi B, Alroy-Preis S, Ash N, Milo R, Huppert A. 2021. BNT162b2 vaccine booster dose protection: a nationwide study from Israel. medRxiv. https://www.medrxiv.org/content/10.1101/2021.08.27.21262679v1. 10.1056/NEJMoa2114255 PMC8461568 34525275

[15] Gao Y, Cai C, Grifoni A, Müller TR, Niessl J, Olofsson A, Humbert M, Hansson L, Österborg A, Bergman P, Chen P, Olsson A, Sandberg JK, Weiskopf D, Price DA, Ljunggren H-G, Karlsson AC, Sette A, Aleman S, Buggert M. 2022. Ancestral SARS-CoV-2-specific T cells cross-recognize the Omicron variant. Nat Med 28:472–476. doi:10.1038/s41591-022-01700-x. 35042228 PMC8938268

[16] Tarke A, Sidney J, Methot N, Yu ED, Zhang Y, Dan JM, Goodwin B, Rubiro P, Sutherland A, Wang E, Frazier A, Ramirez SI, Rawlings SA, Smith DM, da Silva Antunes R, Peters B, Scheuermann RH, Weiskopf D, Crotty S, Grifoni A, Sette A. 2021. Impact of SARS-CoV-2 variants on the total CD4+ and CD8+ T cell reactivity in infected or vaccinated individuals. Cell Rep Med 2:100355. doi:10.1016/j.xcrm.2021.100355. 34230917 PMC8249675

[17] Sieber J, Mayer M, Schmidthaler K, Kopanja S, Camp Jv, Popovitsch A, Dwivedi V, Hoz J, Schoof A, Weseslindtner L, Szépfalusi Z, Stiasny K, Aberle JH. 2022. Long-lived immunity in SARS-CoV-2-recovered children and its neutralizing capacity against Omicron. Front Immunol 13:882456. doi:10.3389/fimmu.2022.882456. 35663948 PMC9157051

[18] Vargas L, Valdivieso N, Tempio F, Simon V, Sauma D, Valenzuela L, Beltrán C, Castillo-Delgado L, Contreras-Benavides X, Acevedo ML, Acevedo J, Gonzalez RI, Valiente-Echeverría F, Soto-Rifo R, Rosemblatt M, Lopez M, Osorio F, Bono MR. 2022. Serological study of CoronaVac vaccine and booster doses in Chile: immunogenicity and persistence of anti-SARS-CoV-2 spike antibodies. BMC Med 20:216. doi:10.1186/s12916-022-02406-0. 35676738 PMC9177225

[19] World Medical Association. 2013. World Medical Association Declaration of Helsinki: ethical principles for medical research involving human subjects. JAMA 310:2191–2194. doi:10.1001/jama.2013.281053. 24141714

[20] Irsara C, Egger AE, Prokop W, Nairz M, Loacker L, Sahanic S, Pizzini A, Sonnweber T, Holzer B, Mayer W, Schennach H, Loeffler-Ragg J, Bellmann-Weiler R, Hartmann B, Tancevski I, Weiss G, Binder CJ, Anliker M, Griesmacher A, Hoermann G. 2021. Clinical validation of the Siemens quantitative SARS-CoV-2 spike IgG assay (sCOVG) reveals improved sensitivity and a good correlation with virus neutralization titers. Clin Chem Lab Med 59:1453–1462. doi:10.1515/cclm-2021-0214. 33837679

[21] Giavarina D, Carta M. 2022. Improvements and limits of anti SARS-CoV-2 antibodies assays by WHO (NIBSC 20/136) standardization. Diagnosis 9:274–279. doi:10.1515/dx-2021-0126. 34851563

[22] Acevedo ML, Gaete-Argel A, Alonso-Palomares L, de Oca MM, Bustamante A, Gaggero A, Paredes F, Cortes CP, Pantano S, Martínez-Valdebenito C, Angulo J, le Corre N, Ferrés M, Navarrete MA, Valiente-Echeverría F, Soto-Rifo R. 2022. Differential neutralizing antibody responses elicited by CoronaVac and BNT162b2 against SARS-CoV-2 Lambda in Chile. Nat Microbiol 7:524–529. doi:10.1038/s41564-022-01092-1. 35365787

[23] Grifoni A, Weiskopf D, Ramirez SI, Mateus J, Dan JM, Moderbacher CR, Rawlings SA, Sutherland A, Premkumar L, Jadi RS, Marrama D, de Silva AM, Frazier A, Carlin AF, Greenbaum JA, Peters B, Krammer F, Smith DM, Crotty S, Sette A. 2020. Targets of T cell responses to SARS-CoV-2 coronavirus in humans with COVID-19 disease and unexposed individuals. Cell 181:1489–1501.e15. doi:10.1016/j.cell.2020.05.015. 32473127 PMC7237901

