# Supplementary material for: Inactivated Vaccine-Induced SARS-CoV-2 Variant-Specific Immunity in Children
Source: mBio. 2022 Nov 16;13(6):e01311-22. doi: 10.1128/mbio.01311-22 (PMC9765711; doi:10.1128/mbio.01311-22)
Supplement: TABLE S1 [file mbio.01311-22-st001.pdf]

Supplementary Table 1. Frequency of local and systemic immediate adverse events by dose and age group.

Frequency of local immediate adverse events by dose and age group.

	Dose 1 (n=963)	Dose 2 (n=624)
**Pain**		
3-11 years	33 (3.8)	9 (1.7)
12-17 years	2 (2.2)	7 (8.2)
**Redness**		
3-11 years	20 (2.3)	3 (0.6)
12-17 years	0	0
**Induration**		
3-11 years	5 (0.6)	0
12-17 years	1 (1.1)	0
**Pruritus**		
3-11 years	2 (0.2)	0
12-17 years	0	1 (1.2)
**Swelling**		
3-11 years	0	0
12-17 years	1 (1.1)	0
**Other**		
3-11 years	1 (0.1)	1 (0.2)
12-17 years	0	0

Frequency of systemic immediate adverse events by dose and age group.

**Headache**		
3-11 years	6 (0.7)	1 (0.2)
12-17 years	2 (2.2)	1 (1.2)
**Fatigue**		
3-11 years	4 (0.5)	1 (0.2)
12-17 years	0	0

	**Muscle pain**	
3-11 years	1 (0.1)	0
12-17 years	0	1 (1.2)
	**Nausea**	
3-11 years	1 (0.1)	0
12-17 years	0	0
	**Skin or mucosa abnormality**	
3-11 years	0	0
12-17 years	0	1* (1.2)

* **Generalized pruritus, without rash or other symptoms, mild and self-limited**

- *Total volunteers for first dose (n=963): 3-11 years (n=873) and 12-17 years (n=90)*
- *Total volunteers for second dose (n=624): 3-11 years (n=539) and 12-17 years (n=85)*
-
