# Supplementary material for: Inactivated Vaccine-Induced SARS-CoV-2 Variant-Specific Immunity in Children
Source: mBio. 2022 Nov 16;13(6):e01311-22. doi: 10.1128/mbio.01311-22 (PMC9765711; doi:10.1128/mbio.01311-22)
Supplement: TABLE S2 [file mbio.01311-22-st002.pdf]

Supplementary Table 2. Description of allergic reactions following vaccination in the 3-11 age group

	Allergic reaction	Timing	Severity	Classification by investigator
**Subject 1**	Left arm rash (same arm of inoculation)	Three days after 1st dose, lasting seven days	Grade 1 in severity (mild)	Possible
**Subject 2**	Rash around mouth and nose	One day after 1st dose and lasted three days	Grade 1 in severity (mild)	Not related
**Subject 3**	Rash in her/his back	Three days after 1st dose and last two days	Grade 1 in severity (mild)	Not related
**Subject 4**	Rash and pruritus in both arms	Eighteen days after 1st dose, lasting one day	Grade 1 in severity (mild)	Not related
**Subject 5**	This subject presented two allergic reactions<br>a) Rash in her/his face after visiting a farm<br>b) Rash in face and arms	a) Eighteen days after 1st dose lasting seven days.<br>b) Three days after 1st dose, lasting one day without any medication	a) Grade 1 in severity (mild)<br>b) Grade 1 in severity (mild)	a) Not related<br>b) Probable
**Subject 6**	The subject has antecedents of atopy and has already facial rash before vaccination but an exacerbation of this rash in her/his face, trunk and arms occurred after vaccination	Three days after 1st dose. The duration is unknown	Grade 2 in severity (moderate)	Probable
**Subject 7**	Genital and perianal rash	Two days after 1st dose. The duration is unknown	Grade 2 in severity (moderate)	Unlikely
**Subject 8**	The subject presented pruritic papules on both arms, which require medication with corticosteroids and antihistamines. This episode coincides with the arrival of a new pet in the house	Seven days after 2nd dose, lasting 17 days, needing medication	Grade 2 in severity (moderate)	Unlikely
