# Supplementary figures and images for: Inactivated Vaccine-Induced SARS-CoV-2 Variant-Specific Immunity in Children
Source: mBio. 2022 Nov 16;13(6):e01311-22. doi: 10.1128/mbio.01311-22 (PMC9765711; doi:10.1128/mbio.01311-22)

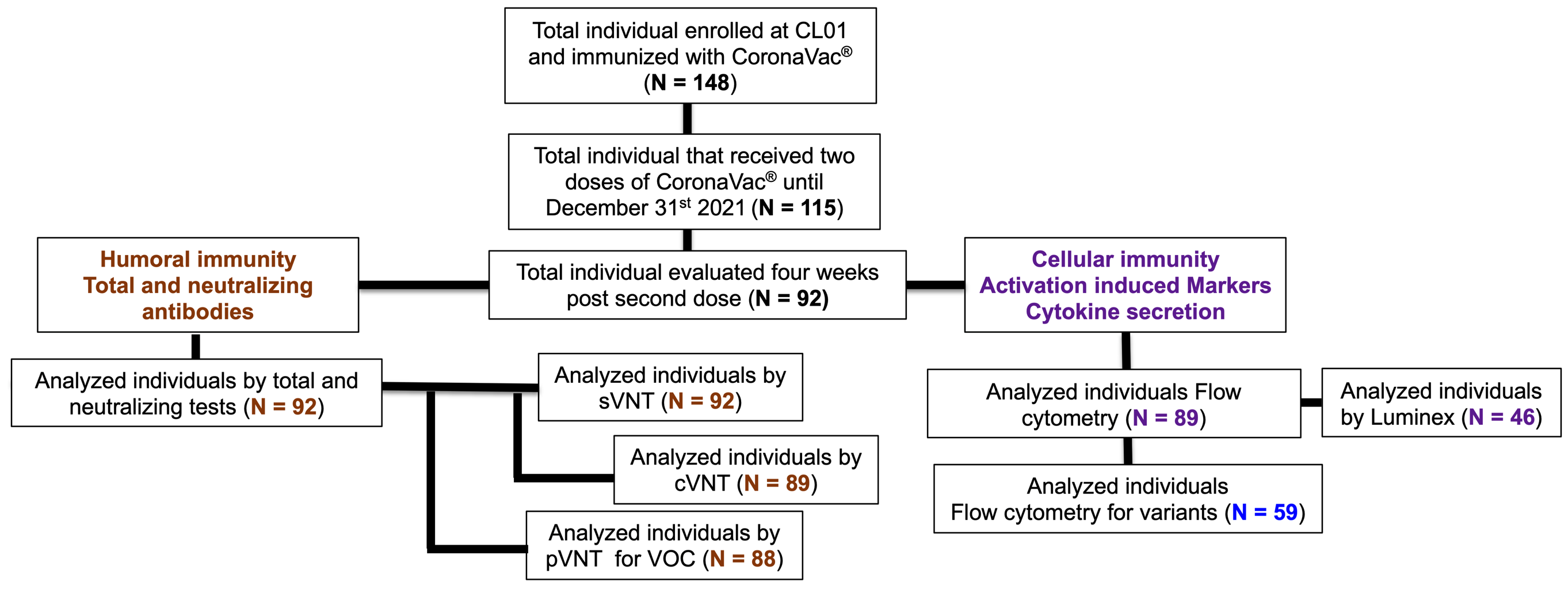

Supplement: FIG S1 [file mbio.01311-22-sf001.tif]

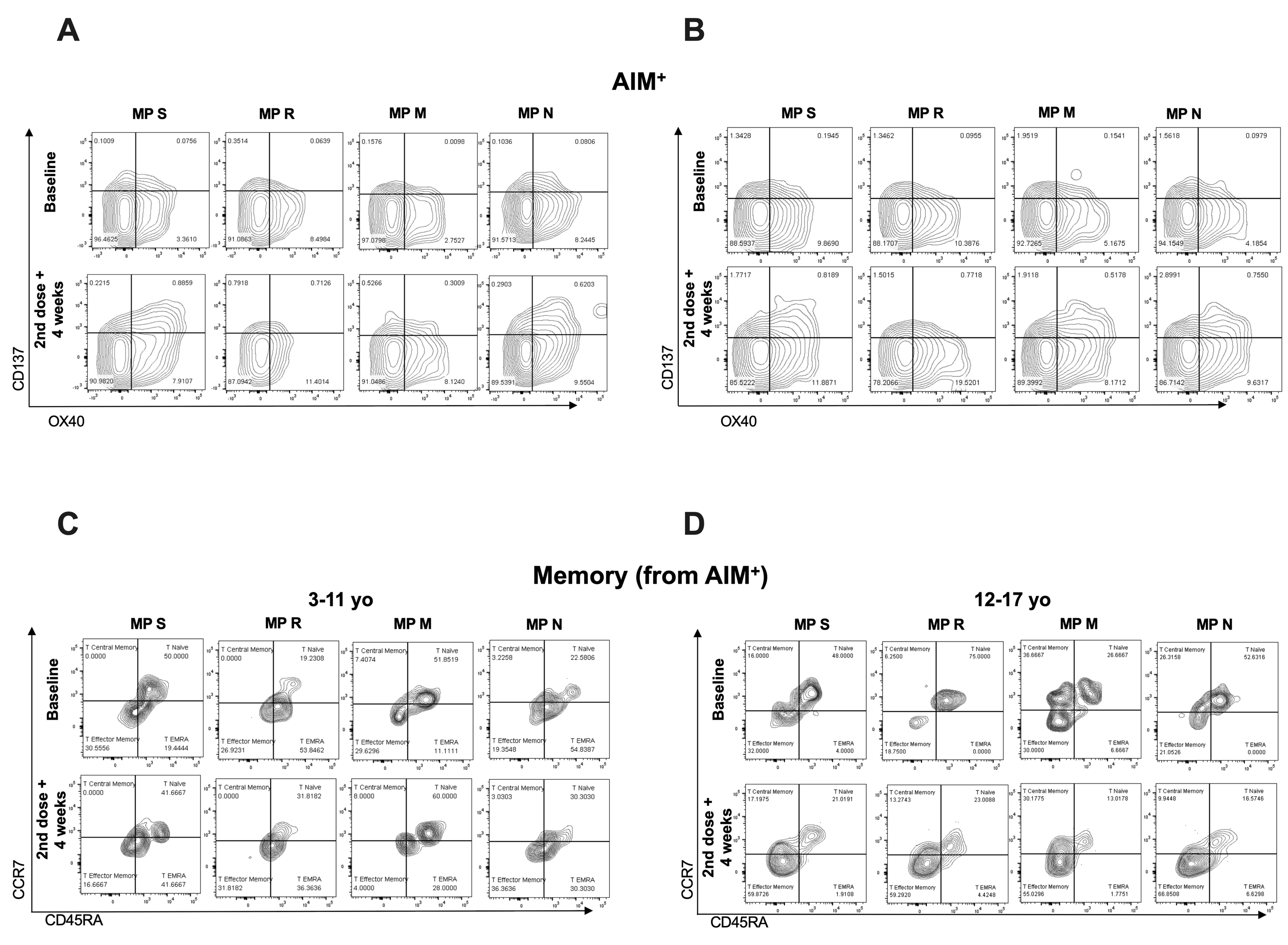

Supplement: FIG S2 [file mbio.01311-22-sf002.tif]

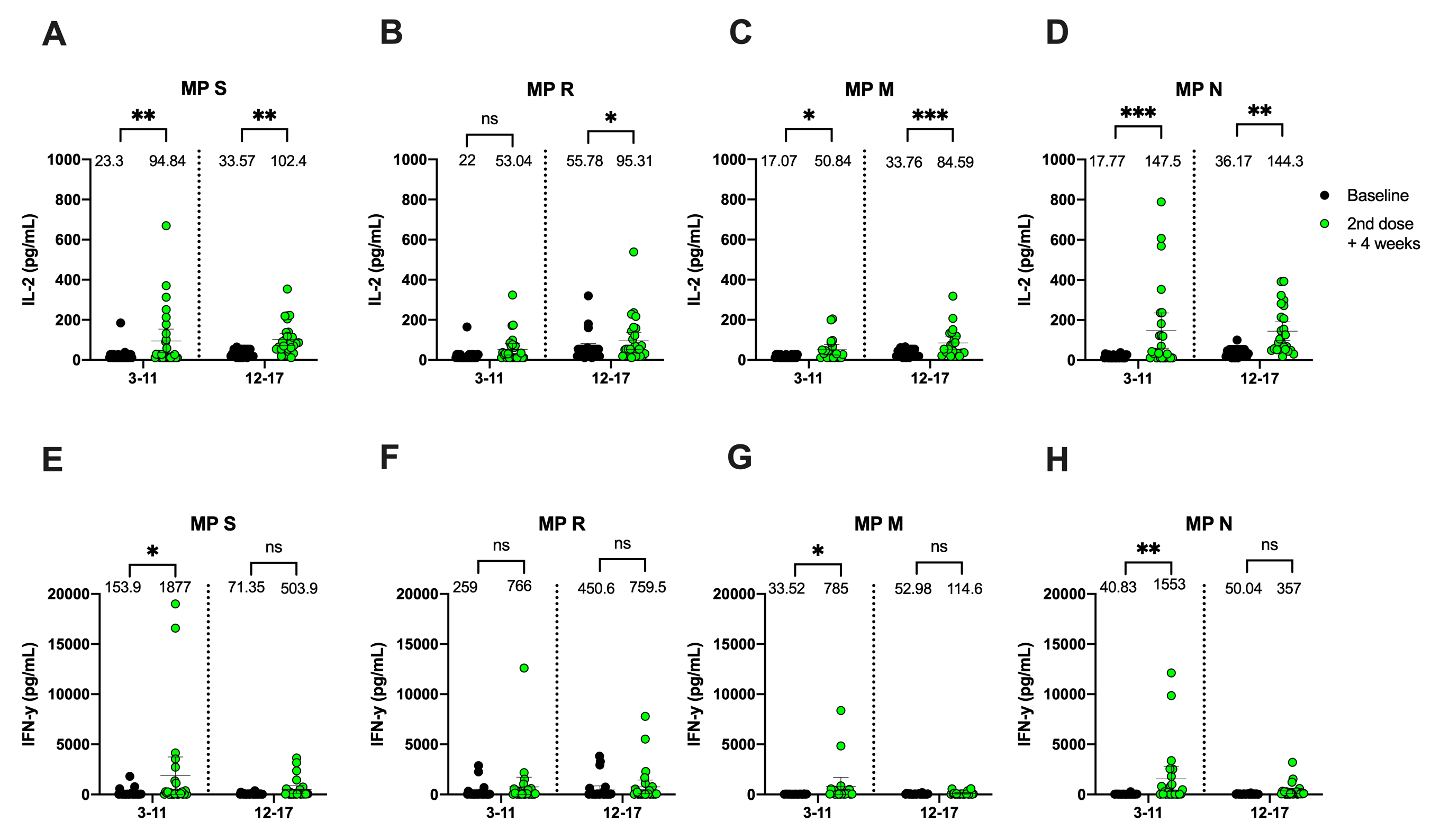

Supplement: FIG S3 [file mbio.01311-22-sf003.tif]

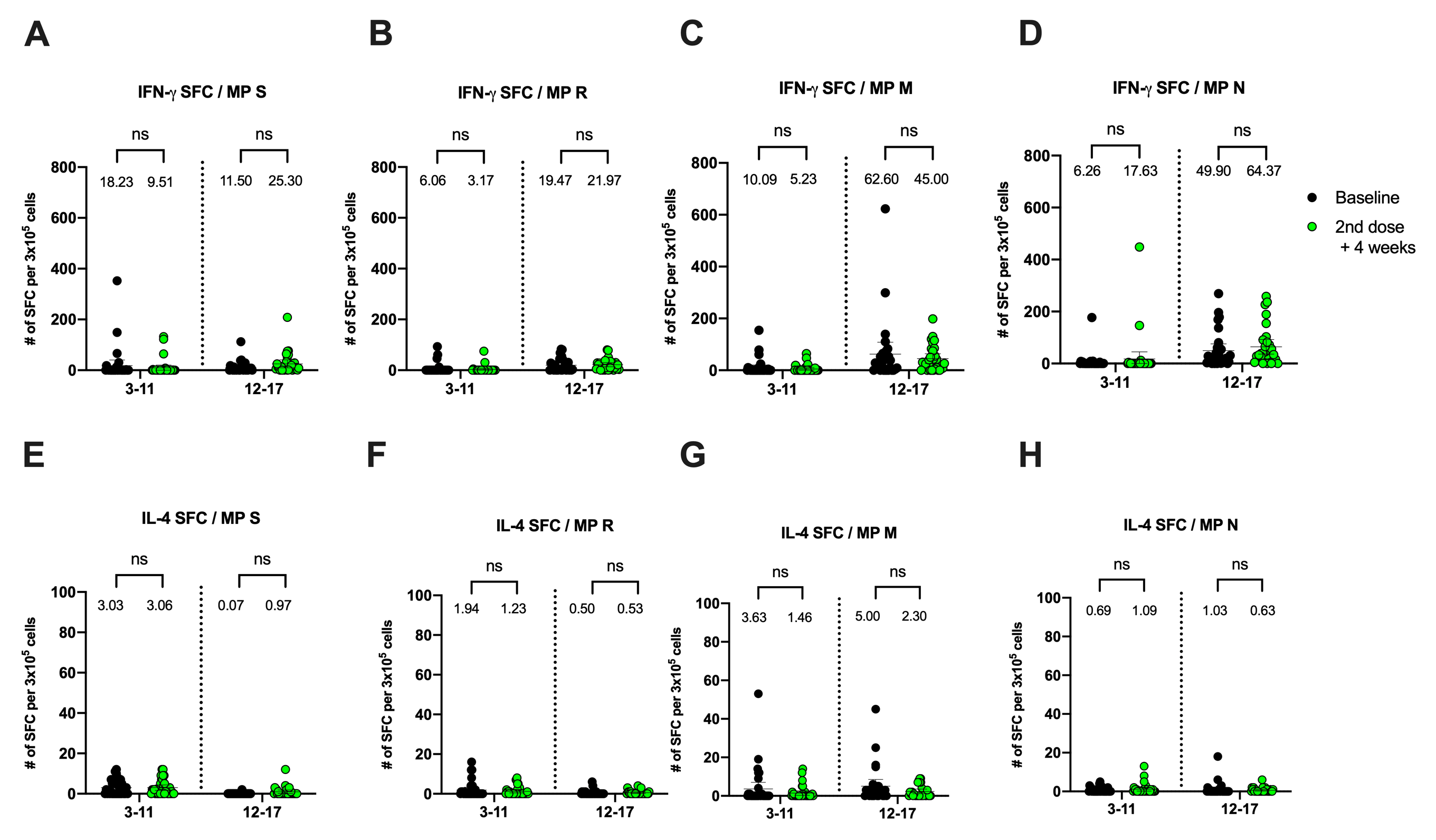

Supplement: FIG S4 [file mbio.01311-22-sf004.tif]

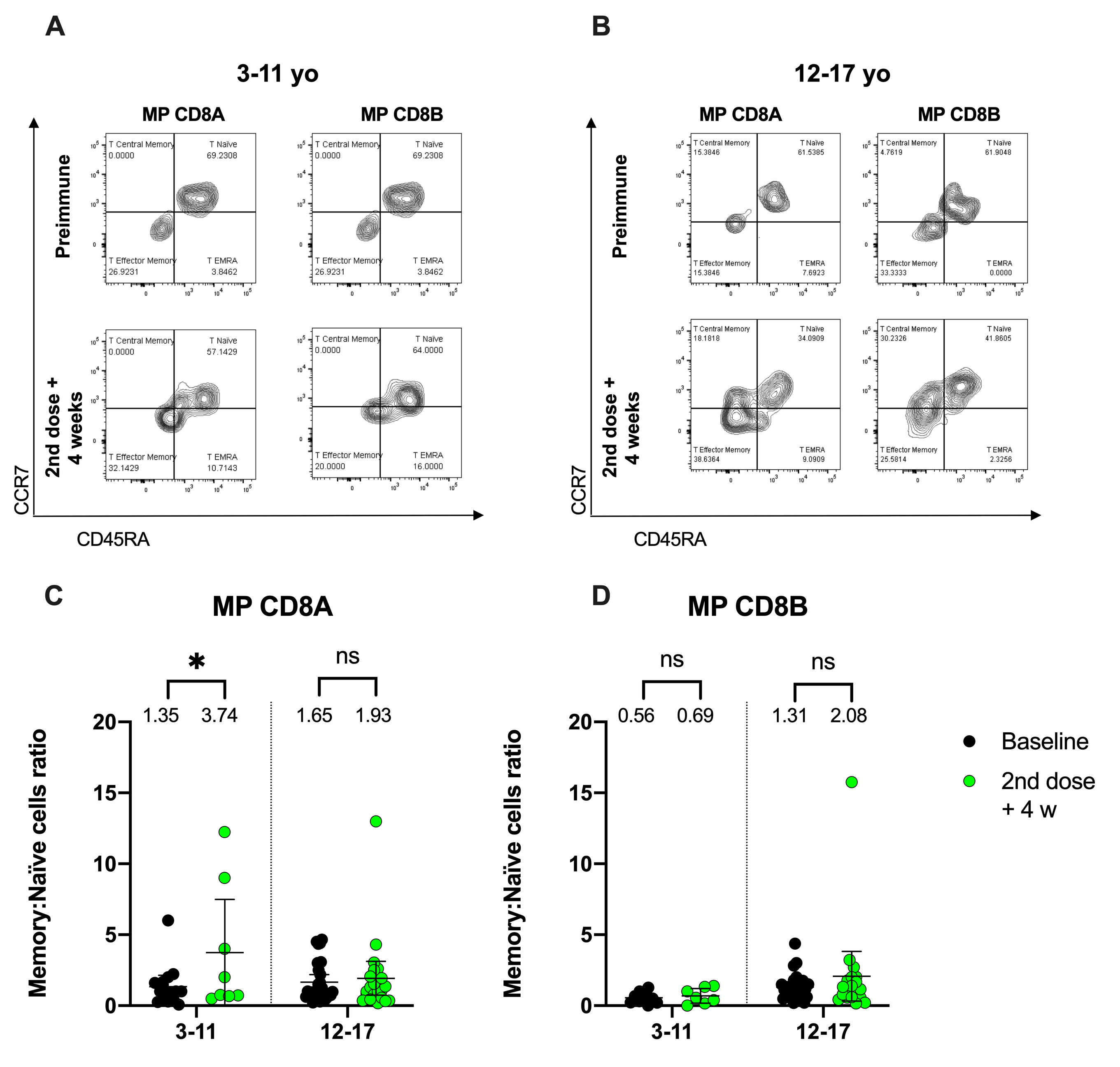

Supplement: FIG S5 [file mbio.01311-22-sf005.tif]
