# Supplementary material for: Inactivated Vaccine-Induced SARS-CoV-2 Variant-Specific Immunity in Children
Source: mBio. 2022 Nov 16;13(6):e01311-22. doi: 10.1128/mbio.01311-22 (PMC9765711; doi:10.1128/mbio.01311-22)
Supplement: TABLE S3 [file mbio.01311-22-st003.pdf]

Supplementary Table 3: Baseline characteristics of 92 participants in immunogenicity group.

	3-11 years old group n=55	12-17 years old group n=37
**Age years, mean (SD)**	7,73 (2,77)	13.7 (1.62)
**Gender female, n (%)**	31 (56,4%)	21 (56,8%)
**With Co-morbidities, n (%)**	35 (63,6%)	24 (64,9%)
**Obesity, n**	2	4
**ENT[a] surgery, n**	13	6
**Mental health[b], n**	5	7
**Drugs allergy, n**	0	4
**Atopy[c], n**	20	9
**Asthma, n**	6	4
**Surgeries, n**	0	8
**Chromosomopathies[d], n**	2	0
**Others, n**	15	12

[a]ear, nose and throat
[b]anxiety disorders, ADHD, ASD
[c]allergic rhinitis -atopic dermatitis
[d] Down Syndrome and Turner Syndrome
