# Supplementary material for: Inactivated Vaccine-Induced SARS-CoV-2 Variant-Specific Immunity in Children
Source: mBio. 2022 Nov 16;13(6):e01311-22. doi: 10.1128/mbio.01311-22 (PMC9765711; doi:10.1128/mbio.01311-22)
Supplement: TABLE S4 [file mbio.01311-22-st004.pdf]

Supplementary Table 4A: Seropositivity rates, Geometric Mean Units (GMU) or titers (GMT) of circulating antibodies against SARS-CoV-2.

		Age Group	
**Test**	**Parameter**	**3-11**	**12-17**
Total IgG anti S1-SARS-CoV-2	Seropositivity n/N	24/25	34/36
	(%)	96	94.5
	GMU	964.9	680.6
	95% CI	503-1850	371-1245
Neutralizing antibodies (sVNT)	Seropositivity n/N	55/55	37/37
	(%)	100	100
	GMU	999.8	492.2
	95% CI	565.8-898.8	342-708.3
Neutralizing antibodies (cVNT)	Seropositivity n/N	27/27	30/34
	(%)	100	88.2
	GMT	146.3	38.6
	95% CI	93.96-227.6	21.1-70.7

Supplementary Table 4B: Seropositivity rates, Geometric Mean titers (GMT) of circulating antibodies against variant of concern of SARS-CoV-2.

Test	Parameter	Variant		
		D614G	Delta	Omicron
Neutralizing antibodies (pVNT)	Seropositivity n/N	88/88	86/88	40/88
	(%)	100	97.7	45.5
	GMT	265.4	141.6	16.81
	95% CI	213.1-330.5	113.6-176.5	14.0-20.3
