# Supplementary material for: Inactivated Vaccine-Induced SARS-CoV-2 Variant-Specific Immunity in Children
Source: mBio. 2022 Nov 16;13(6):e01311-22. doi: 10.1128/mbio.01311-22 (PMC9765711; doi:10.1128/mbio.01311-22)
Supplement: TEXT S1 [file mbio.01311-22-s0001.docx]

**Supplementary information**

**Materials and methods**

**Study design**

In Chile, this trial has been conducted at eleven different sites, eight in the center of the country (seven in Santiago and one in Valparaiso), two in the South (Puerto Montt and Valdivia), and one in the North (Antofagasta) of Chile.

**Inclusion and Exclusion Criteria**

**Inclusion Criteria**

1. Healthy children and adolescents aged 6 months to 17 years;

2. The participants and/or their guardians are able to understand and sign the informed consent voluntarily (in accordance with the local regulations);

3. Able to comply with study procedures based on the assessment of the Investigator;

4. Female participants of childbearing potential (post-menarche girls or in accordance with the local standard of care) may be enrolled in the study if the participant fulfills all the following criteria:

• Has a negative pregnancy test on the day of the first dose (Day 0).

• Has practiced adequate contraception or has abstained from all activities that could result in pregnancy for at least 28 days prior to the first dose (Day 0).

• Has agreed to continue adequate contraception through 3 months following the second dose (Day 28).

• Is not currently breastfeeding.

5. Must be willing to provide verifiable identification (in accordance with the local regulations), has means to be contacted and to contact the investigator during the study.

**Exclusion Criteria**

Participants are excluded from the study if any of the following criteria apply:

- History of confirmed infection of SARS CoV-2 prior to randomization;
- History of contact with person infected with SARS-CoV-2 (has a positive nucleic acid test or an antigen test) within 14 days prior to randomization;
- Prior administration of an investigational or licensed coronavirus vaccine or current/planned simultaneous participation in another interventional study to prevent or treat COVID-19;
- Allergy to vaccines or vaccine/placebo ingredients, and serious adverse reactions to vaccines, such as urticaria, dyspnea, angioneuroedema;
- Personal or first-grade relative (siblings) history of multisystem inflammatory disease in children (MIS-C);
- Significant chronic illnesses that, in the opinion of the investigator, is at a stage where it might interfere with trial conduct or completion (may include, but are not limited to cardiovascular disease, liver or kidney disorders, respiratory illnesses)
- Significant chronic central nervous system diseases or neuromuscular disorders, psychosis or severe cognitive behavioral disorder, in the opinion of the investigator, including epilepsy, autism spectrum disorder, intellectual disabilities (excluding Down Syndrome);
- Acute central nervous system diseases such as encephalitis/myelitis, acute disseminating encephalomyelitis, and related disorders;
- History of autoimmune and/or haematological diseases (including but not limited to systemic lupus erythematosus, thyroidectomy, autoimmune thyroid disease, any form of malignant tumor, asplenia, functional asplenia, or splenectomy resulting from any condition); well controlled type I diabetes mellitus is allowed;
- History of bleeding disorders (e.g. factor deficiency, coagulopathy or platelet disorder), or prior history of significant bleeding or bruising following IM injections or venipuncture;
- Immunosuppressive therapy (systemic corticoid therapy, e.g. prednisone ≥2 mg/Kg/d or ≥20 mg/day for >14 days), cytotoxic therapy (antineoplastic chemotherapy, radiation therapy), (excluding topical or aerosol corticosteroid therapy) in the past 6 months;
- Receipt of blood products or immunoglobulins in the past 3 months;
- Receipt of other investigational drugs in the past 30 days;
- Receipt of attenuated live vaccines in the past 14 days;
- Receipt of inactivated or subunit vaccines in the past 7 days;
- Emerging of chronic diseases or acute exacerbation of stable chronic diseases (including but not limited to asthma, migraine, gastrointestinal disorder, etc.) prior to randomization;
- Acute febrile illness with oral temperature >37.6°C or axillary temperature >37.4°C on the day of vaccination (refer to section 7.1 Delay/Discontinuation of Study Vaccination); enrollment could be considered if the fever is absent for 72 hours;
- Any confirmed or suspected human immunodeficiency virus (HIV) infection;
- Children in care or under a court order;

According to the investigator's judgment, the subject has any other factors that might interfere with the results of the clinical trial or pose additional risk to the subject due to participation in the study.

**Study population and outcomes**

For all participants, immediate AE (30 min post-vaccination) and serious adverse events (SAE), and adverse events of special interest (AESI) were recorded. The study aims were to evaluate the immunogenicity of CoronaVac^®^ in a subgroup of participants 4 weeks after 2 doses and the frequency of solicited immediate (first 30 min post-dose) and non-immediate adverse events (AEs) that occur during seven days after each dose, stratified by age group (3-11 and 12-17 years old), and the frequency of SAE/AESI and any other AE occurring 28 days after each dose, and the frequency of any SAE/AESI occurring 12 months after the second dose.

**Sample collection**

Blood samples were obtained in heparinized tubes before administration of the first dose (pre-immune) and four weeks after the second dose. Samples were used to obtain plasma and peripheral blood mononuclear cells (PBMCs) and stored at -80ºC (plasma) and -170ºC (PBMCs) until humoral and cellular immunity analyses were performed. The sample size included in each experimental analysis is described in **Suppl. Figure 1**.

**Antibodies evaluation**

IgG anti-S1-RBD of SARS-CoV-2 were tested using ADVIA Centaur® XP SARS-CoV-2 IgG (sCOVG, Siemens) (20,21), an automated two-step sandwich antibody-binding immunoassays using indirect chemiluminescence. sCOVG was used for quantitative detection expressed in BAU/mL after interpolating the WHO standard NIBSC code 20/136 calibration.

For the surrogate virus neutralization test (sVNT) (Genscript Cat#L00847-A), two-fold serial dilutions were prepared for each sample, starting at a 4-fold until reaching a 512-fold dilution, and the assay was performed according to manufacturers’ instructions (2). The end titer of neutralizing antibodies was assigned as the last fold dilution that displayed a cut-off over 30% of inhibition. Samples with undetermined concentration at the lowest dilution tested (1:4) were assigned the lower limit of quantification (16.4 IU).

Conventional virus neutralization tests (cVNT) were performed as previously reported (2). Briefly, Vero E6 cells were infected with a SARS-CoV-2 strain obtained by viral isolation in tissue cultures (33782CL-SARS-CoV-2 strain, D614G variant). Neutralization assays were carried out by the reduction of cytopathic effect (CPE) in Vero E6 cells (ATCC CRL-1586). The titer of neutralizing antibodies was defined as the highest plasma dilution that neutralized virus infection, at which the CPE was absent as compared with the virus control wells (cells with CPE). Vero E6 cells were seeded in 96-well plates (4×10^4^ cells/well). For neutralization assays, 100 µL of 33782CL-SARS-CoV-2 (at a dose of 100 TCID_50_) were incubated with serial dilutions of heat-inactivated sera samples from participants (dilutions of 1:4, 1:8, 1:16, 1:32, 1:64, 1:128, 1:256, and 1:512) from participants for 1h at 37 °C. Cytopathic effect on Vero E6 cells was analyzed seven days after infection.

A pseudotyped virus neutralization test (pVNT) assay was performed to assess the neutralization capacity of the antibodies against SARS-CoV-2 variants of concern (VOC). As previously reported (12), an HIV-1 backbone expressing firefly luciferase as a reporter gene and pseudotyped with the SARS-CoV-2 spike glycoproteins (HIV-1-SΔ19) from lineage B.1 (D614G) or variants Delta (T19R, del157/158, L452R, T478K, D614G, P681R, D950N) and Omicron (A67V, ∆H69-V70, T95I, Y145D, ∆G142 -V143-Y144, ∆N211, EPE 213-214, G339D, S371L, S373P, S375F, K417N, N440K, G446S, S477N, T478K, E484A, Q493R, G496S, Q498R, N501Y, T547K, D614G, H655Y, N679K, P681H, N764K, N865K, Q954H, N969K, L981F) was prepared as previously described (22). Plasma samples were two-fold diluted, starting at 1:10 or 1:4, and the estimation of the ID80 was obtained using a 4-parameter nonlinear regression curve fit measured as the percent of neutralization determined by the difference in average relative light units (RLU) between test samples and pseudotyped virus controls. Data analyses and statistical analyses were carried out using GraphPad Prism v9.

**Evaluation of cellular immune response**

In order to assess the cellular immune response, PBMCs of sixty participants were stimulated with six Mega Pools (MPs) of peptides derived from the proteome of SARS-CoV-2, including peptides from the S protein (MP-S), the remaining proteins of the viral particle (excluding S protein peptides) (MP-R), peptides from the proteins M (Miltenyi, Cat#130-126-702) and N (Miltenyi, Cat#130-126-698) and MHC-I restricted peptides from the whole proteome of SARS-CoV-2 (MP-CD8-A and MP-CD8-B).

Supernatants from PBMCs stimulated with SARS-CoV-2 MPs for 20h were evaluated using the Luminex^®^ technology (R&D systems, USA) to assess IL-2 and IFN-γ production. Briefly, supernatants of samples stored at -80ºC were thawed at room temperature and diluted 1:2 before analysis. After 2 h incubation with spectrally encoded beads, coated with analyte-specific biotinylated primary antibodies, the samples were incubated with streptavidin R-phycoerythrin and analyzed using a Luminex 200 xMap multiplex system (Luminex Corporation, Austin, TX). According to the manufacturer’s instruction the detection limit for the cytokines measured ranged from 4.2 to 13,390 pg/mL.
